# Injectable Arnebia Euchroma Polysaccharide-Based Hydrogel as CpG Oligonucleotide Delivery System with Dual Immunomodulatory Activities

**DOI:** 10.3390/gels12080703

**Published:** 2026-08-05

**Authors:** Chengxiang Xiao, Man Zhang, Mu Dan, Yaru Hu, Peng Zhao, Sarangowa Ochir, Wenming Bai, Surina Bo

**Affiliations:** 1 College of Pharmacy, Inner Mongolia Medical University, Jinshan Development Zone, Hohhot 010110, China; 2 College of Medical, Shandong Xiehe University, Guodian Development Zone, Jinan 250109, China; 3 Department of Chemical and Biological Engineering, School of Engineering and Technology, National University of Mongolia, Ulaanbaatar 14201, Mongolia; 4 Academy of Mongolian Medicine, Inner Mongolia Medical University, Jinshan Development Zone, Hohhot 010110, China

**Keywords:** immunostimulatory hydrogel, *Arnebia euchroma* polysaccharide, CpG oligonucleotide delivery, dual immunomodulation

## Abstract

This study developed novel immunostimulatory hydrogels composed of aldehyde-modified *Arnebia euchroma* polysaccharide (oxidized ARP, O-ARP) and gelatin (GE) for oligonucleotide delivery. Structural characterization of three O-ARP derivatives with varying degrees of oxidation confirmed reduced molecular weight, a preserved yet altered molar ratio of monosaccharide composition, and triple-helical conformation. Hydrogels (GE-O1, GE-O2, GE-O3) were formed via a Schiff base reaction between aldehyde and amino groups. Molecular dynamics simulations showed that O-ARP and gelatin can form a stable three-dimensional (3D) network via hydrogen bonding and van der Waals interactions. In vitro studies demonstrated that both the O1-ARP derivative and GE-O1 hydrogel significantly improved RAW 264.7 macrophage viability, phagocytosis, NO production, and pro-inflammatory cytokine secretion, including IL-6, IL-1β, and TNF-α. The cationic GE-O1 hydrogel efficiently loaded anionic CpG oligonucleotides (CpG-ODN 1862) via electrostatic interaction, forming GE-O1-CpG complexes and promoting cellular uptake. Importantly, the GE-O1-CpG complex exhibited superior immunomodulatory effects compared with either GE-O1 or CpG alone, indicating a synergistic dual immunostimulatory response. In vivo studies confirmed the biosafety of GE-O1-CpG. Using OVA as a model antigen, GE-O1-CpG/OVA enhanced both humoral and cellular immune responses. These findings support GE-O1-CpG hydrogels as potential system for combined immunomodulation and nucleotide delivery.

## 1. Introduction

Among the numerous potential nucleic acid therapeutics, microbial CpG DNA containing unmethylated CpG motifs (CpG ODNs) is a candidate that can stimulate the activation or proliferation of a wide range of immune cells with strong functional capacity [1]. Hemmi et al. discovered that Toll-like receptor 9 (TLR9) is essential for CpG ODN-mediated immune activation and may act as its intracellular receptor [2]. Upon CpG stimulation, TLR9 recruits the adaptor protein myeloid differentiation factor (MyD88), which phosphorylates IL-1 receptor-associated kinase 4 (IRAK-4) and interacts with tumor necrosis factor receptor-associated factor 6 (TRAF6), as well as IL-6, IL-10, IL-12, type I IFNs, TRAF6, and TNF-α [3,4]. Clinical studies have shown that CpG motifs can function as adjuvants in vaccines against infectious pathogens, tumor antigens, and allergens [5]. However, their short circulation period in biofluids is a significant disadvantage due to the activity of nucleases [6]. An effective delivery system is crucial for the clinical application of therapeutic oligonucleotides.

Numerous synthetic polymer carriers—including polyethyleneimine (PEI) [7], poly (N, N-dimethyl aminoethyl methacrylate) (PDMAEMA) [8], poly(L-lysine) (PLL) [9], and poly(amidoamine) (PAMAM) [10]—have been extensively investigated for nucleic acid transport. Yet these polymeric vectors face persistent drawbacks, such as high cytotoxicity, low transfection efficiency, poor serum stability in vivo, and limited targeting specificity. Consequently, the creation of novel, biocompatible, and efficient polymeric vectors with intrinsic targeting capacity remains a significant challenge.

Polysaccharide-based hydrogels represent ideal drug carriers due to their abundance, biocompatibility, biodegradability, native biodiversity, and nontoxicity, making them highly attractive for pharmaceutical applications. Natural polysaccharides contain multiple modifiable functional groups that can be chemically engineered to facilitate cross-linking with other materials, enabling the formation of hydrogels with excellent material properties and the preservation of biological activities. Among these chemical strategies, the aldehyde functionalization of polysaccharides has garnered significant attention. Oxidative modification of natural polysaccharides—such as oxidized Bletilla striata polysaccharide [11], oxidized pullulan [12], oxidized pectin [13], and oxidized alginate [14]—has been widely applied to impart gelation properties. The degree of cross-linking and overall hydrogel performance are directly linked to the aldehyde group content in oxidized polysaccharides [15]. For example, Zhang et al. fabricated a hydrogel using oxidized Bletilla striata polysaccharide (BSP) and cationic gelatin via Schiff base reactions, where the mechanical strength, pore structure, swelling behavior, degradability, and antioxidant capacity of the material depended on the aldehyde concentration of the oxidized BSP, making it suitable for diverse wound repair applications [11].

The root of *Arnebia euchroma* (Royle) Johnst., a traditional herbal medicine in Tibet and Mongolia, was first documented in Shen Nong’s *Materia Medica* [16]. It has been traditionally used to cool the blood, promote circulation, clear heat, and remove toxins [17]. Polysaccharides are regarded as the key active constituents of A. euchroma. In our previous work, we purified a homogenous polysaccharide, ARP, composed of (1→6)-linked galactose, (1→3)-linked arabinose (77.3% arabinogalactan), and glucose (12.2%). ARP exhibited significant immunomodulatory effects on mouse splenic lymphocytes and promoted cytokine secretion (IL-6, TNF-α, IL-2, etc.) by macrophages in vitro. We hypothesized that the remarkable immune-modulating activity of ARP is closely related to its high arabinogalactan content [18].

In this study, we proposed the construction of a dynamic injectable hydrogel based on the natural ARP for delivering CpG oligonucleotides with dual immunological activity. The hydrogel was designed using dynamic cross-linkers, where the aldehyde groups of oxidized ARP form Schiff base bonds with the amine groups of gelatin. For this purpose, we prepared three oxidized ARP derivatives with varying degrees of substitution (low, medium, and high) by tuning the oxidation reaction conditions. This strategy was designed to systematically investigate the influence of aldehyde group content on gel properties and to identify the most suitable candidate material for nucleic acid loading. To investigate the behavior and mechanisms of three oxidized polysaccharide hydrogels, their rheological properties, swelling, degradation, and molecular dynamics simulations were analyzed. Additionally, GE-O1 hydrogel was identified as a suitable carrier for CpG ODN 1862. Its in vitro and in vivo immunomodulatory activities, along with those of its CpG-loaded complex, were systematically evaluated. Collectively, these findings provide experimental evidence and a theoretical foundation for applying natural polysaccharide hydrogel/CpG complexes as dual-functional carriers that serve both as immunostimulants and as CpG oligonucleotide delivery vectors.

## 2. Results and Discussion

### 2.1. Physicochemical Properties of Oxidized ARP

**Figure 1:**
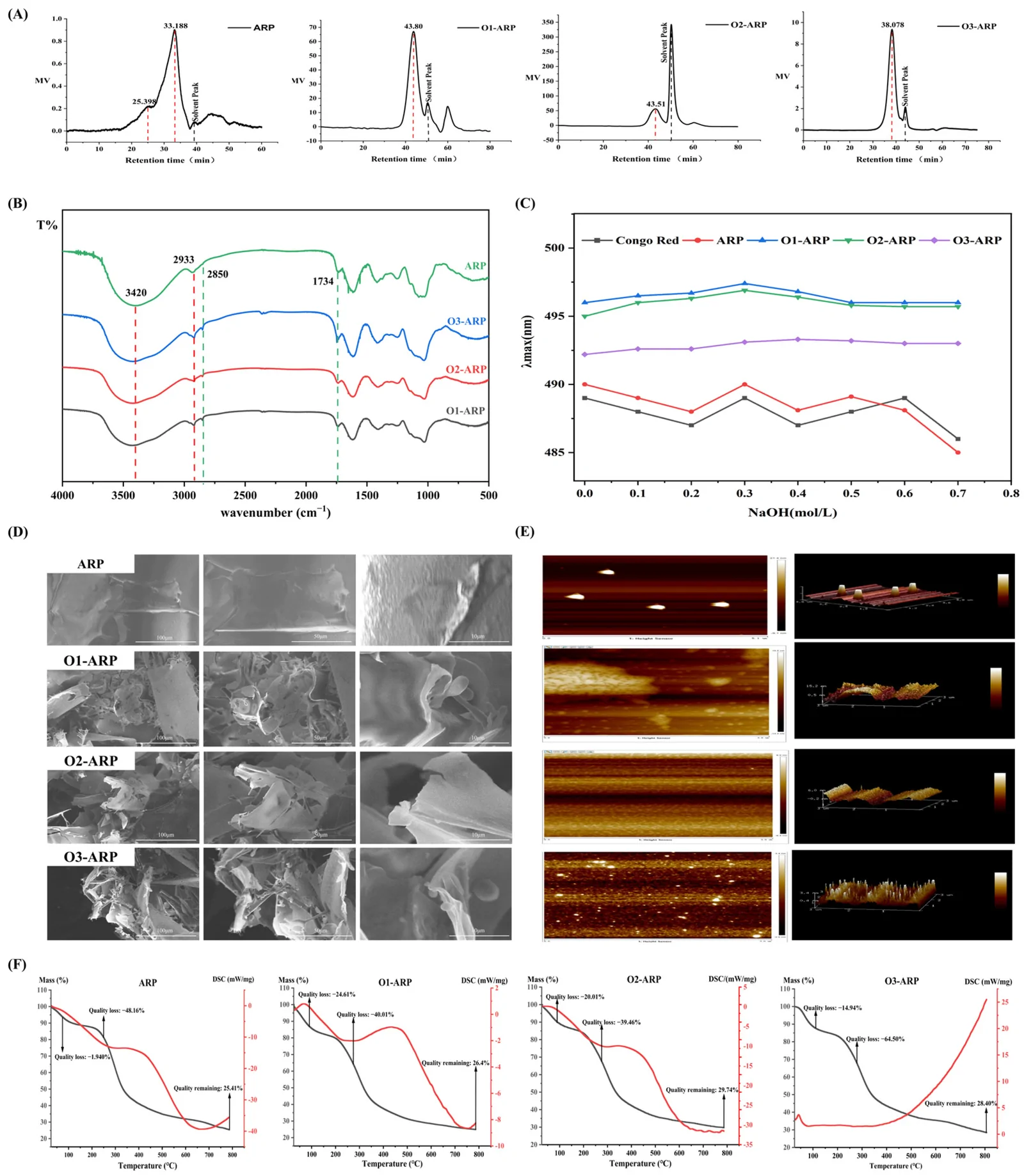
Characterization of ARP and oxidized ARPs. (**A**) Molecular weight measurement of oxidized ARPs by GPC: chromatographs of O1-ARP, O2-ARP, and O3-ARP. (**B**) Fourier transform infrared (FT-IR) spectra. (**C**) Congo red assay. (**D**) Scanning electron microscopy (SEM) images captured at 500×, 1000× and 5000× magnification (left: 500×; middle: 1000×; right: 5000×) to illustrate macromolecular aggregates of ARP and three oxidized polysaccharides. (**E**) AFM images: Left, height images with 5 μm scale; right, 3D images. (**F**) Thermogravimetric analysis curves.

The chemical compositions of the oxidized ARP are summarized in Table 1. Hydroxylamine hydrochloride readily reacts with aldehyde groups of oxidized polysaccharides to form oximes and release HCl, and aldehyde content was determined by acid–base titration. The aldehyde concentrations (-CHO) of O1-ARP, O2-ARP, and O3-ARP were 0.3260, 0.2188, and 0.1900 mol/g, respectively. Based on dextran calibration curves (y = −0.225x + 11.457, R^2^ = 0.9945), the Mw values of O1-ARP, O2-ARP, and O3-ARP were 0.6468 × 10^4^, 0.7627 × 10^4^, and 0.9928 × 10^4^ Da (Figure 1A), which were notably lower than that of native ARP (1.21 × 10^4^ Da) [18]. This reduction is attributed to sodium periodate oxidation, which introduces two aldehyde groups per oxidized monomer by cleaving the C2-C3 bond, thereby shortening polysaccharide chains and lowering molecular weight [19]. The polydispersity coefficients (α = Mw/Mn) of O1-ARP, O2-ARP, and O3-ARP were 1.040, 1.038, and 1.008, respectively. An α value close to 1 indicates high uniformity, typically observed in ideal synthetic polymers or fractionated samples, suggesting that ARP forms highly uniform molecules after oxidation.

Oxidative modification introduces aldehyde groups that regulate gelation time and hydrogel strength while conferring responsiveness to environmental stimuli. However, this approach may result in polysaccharide chain degradation, and precise control over reaction extent under strong oxidation conditions remains challenging [20].

The monosaccharide composition analysis was performed via ion chromatography with 11 monosaccharide standards and revealed the eight monosaccharides—Gal, Ara, Glu, GalA, Man, Xyl, GluA, and Fuc—in the native ARP and oxidized ARP derivatives; however, the molar ratio of sugar composition was altered significantly after oxidization (Table 1). Particularly, the amounts of Gal, GalA, and Glu deceased to 21.75, 6.26, and 9.7 in O1-ARP and 22.64, 7.06 and 10.03 in O2-ARP, but only moderate change was observed in O3-ARP (Figure S1).

The above results suggested that the oxidation by sodium periodate may occur primarily on the 1–6 galactose backbone; at the same time, some side-chain-like terminal galactose (Gal-(1→), terminal glucose (Glc-(1→), (1→4,6)-linked glucose and (1→3,4)-linked rhamnose residues may also be oxidized. A similar reduction in total sugar content was observed after oxidation, from 91.00% in ARP to 60.34%, 67.28%, and 70.13% in O1-ARP, O2-ARP, and O3-ARP, respectively. The above results, manifested by the increase in the substitution degrees of ARP, including the decrease in the Mw, total sugar content, and the molar ratio of monosaccharide in O-ARP, maybe due to glycosidic bonds breaking after oxidation. Meanwhile, oxidation reactions are likely to occur predominantly on the galactose ring.

### 2.2. Characterization of the Oxidized ARP

The oxidized polysaccharides were further characterized by FT-IR. As shown in Figure 1B, compared with unmodified ARP, the characteristic peak at 3400 cm^−1^ for the O-H stretching vibration was weakened and became broader in the O-ARP, likely due to the reduction of O-H groups after modification. The peaks of C-H asymmetric and symmetric stretching vibrations at 2900 cm^−1^ and 2800 cm^−1^ were not significantly altered, whereas the peak intensity of the C=O stretching vibration at 1734 cm^−1^ was strengthened, likely due to dialdehyde modification [21].

Congo red was reacted with ARP and O-ARP derivatives, and the shift in λ_max_ wavelength was measured at varying concentrations of NaOH, as depicted in Figure 1C. The O1-ARP and O2-ARP oxidized polysaccharide–Congo red complexes exhibited a significant red shift at 0.4 M NaOH concentration, suggesting the presence of a triple-helix structure in highly oxidized samples. Liu et al. performed selective aldehyde oxidation on the side groups of lentinan triple-helix polysaccharides using the sodium periodate oxidation method, which enhanced their reducibility and cross-linking adhesion properties. Using this modified polysaccharide as a template, they prepared an oxidized lentinan–gold nanocluster complex. This complex exhibited excellent electrochemiluminescent properties, demonstrating improved activity of the polysaccharide after oxidative modification [22]. For arabinoxylan, the λ_max_ of the complex first increases and then gradually stabilizes as the alkali concentration increases, indicating that Congo red can bind to ordered double-helix structures [23].

The surface morphology of the oxidized samples was scanned using a field-emission ambient scanning electron microscope at magnifications of 500×, 1000×, and 5000×. As illustrated in Figure 1D, in comparison to unmodified ARP, the three oxidized ARPs displayed linked lamellar structures that appeared fractured and irregular, with some uneven bumps and depressions on the surfaces, as well as more fragments. At higher magnifications of 1000× and 5000×, the surface of the fragments became rougher, showing many tiny protrusions and holes, and the structure appeared looser. Changes in morphological characteristics in the oxidized polysaccharides are consistent with a decrease in molecular weight and changes in chain aggregation behavior, which were independently confirmed by GPC.

AFM can characterize surface morphology and the spatial distribution of macromolecular polysaccharides at the nanoscale, including chain diameters, lengths, and distributions. From the two-dimensional images, the O-ARP surface showed clear parallel stripes with uniform spacing and small distances between the stripes. The overall surface was relatively flat and regular, with no obvious bumps or particles (Figure 1E, left). The 3D images clearly delineated the structure of the parallel stripes, which formed a regular undulating pattern with minor height differences. The overall surface was very flat and regular, further indicating that the oxidized ARPs have a high degree of order and uniformity at the microscopic scale (Figure 1E, right). This highly ordered and homogeneous surface structure may confer unique properties to the oxidized ARPs. Additionally, the molecular single chains of polysaccharides are generally 0.1–1.0 nm, while the height of the O-ARP samples was greater than 1 nm, indicating that the modified polysaccharide samples are more prone to aggregation in solvents. The triple-helical structure may aggregate based on hydrogen bonding and van der Waals forces, which aligns with the results of the Congo red experiment.

Thermogravimetric differential scanning calorimetry (TG-DSC) was used to study mass loss and thermal transformations during heating in an inert atmosphere [24]. Figure 1F shows the TGA curves of ARP and three oxidized polysaccharides in nitrogen and oxygen environments across the temperature range of 25–800 °C. The thermogravimetric curves (TG, black curves) reflect the mass loss during the heating process, which corresponds to physicochemical changes (e.g., evaporation of water, chemical bond breakage, and structural decomposition) in different temperature intervals; the DSC curves (red curves) indicate the thermal effects (heat absorption/exothermic), helping to determine the type of reaction. The mass loss occurs in three distinct stages. From 25 to 100 °C, the first stage progresses slowly, and the order of mass reduction rate was O1-ARP > O2-ARP > O3-ARP, reflecting the water retention properties of the polysaccharides. The decomposition phase of the polysaccharides occurs between 200 and 400 °C. The order of mass loss rate was O3-ARP > O1-ARP > O2-ARP > ARP. During this stage, periodate oxidation breaks the polysaccharide glycosidic bonds, leading to molecular chain fragmentation. The thermal stability of the main chain is reduced, and the fragmented short-chain structures are more prone to chemical bond breakage (such as C-C and C-O bonds) and oxidative decomposition (such as aldehyde oxidation) at 200–400 °C, releasing a large number of small molecular products. This causes the increased mass loss rate observed in O3-ARP. These findings reflect the effect of modification on the thermal stability of the polysaccharides.

### 2.3. In Vitro Immunomodulatory Activity of the Oxidized ARP

**Figure 2:**
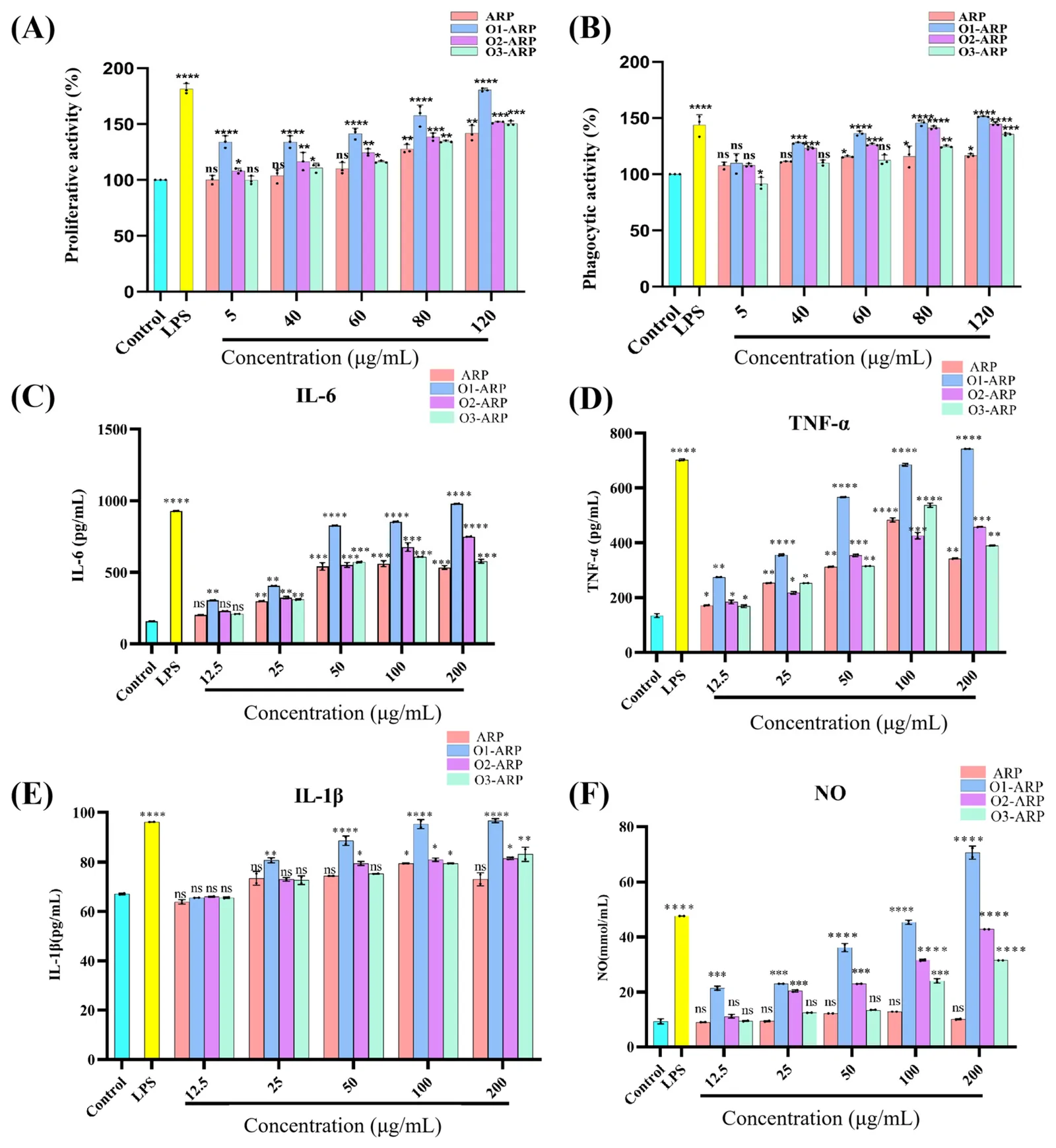
Immunomodulatory activity of ARP, O1-ARP, O2-ARP, and O3-ARP on RAW 264.7 cells in vitro. RAW 264.7 cells were cultured with sample polysaccharides at varying concentrations for 24 h. Macrophage proliferation activity (**A**). Phagocytic activity (**B**). The culture supernatant collected was assayed for the levels of IL-6 (**C**), TNF-α (**D**) IL-1β (**E**), and NO (**F**). LPS was used as the positive control. * *p* < 0.05, ** *p* < 0.01, *** *p* < 0.001 and **** *p* < 0.0001 versus the untreated control; ns indicates no significant difference (*p* ≥ 0.05). Results are expressed as the mean ± SD (*n* = 3).

To estimate the immunomodulatory activity of ARP after oxidative modification, the CCK-8 assay was applied to evaluate the cytotoxicity and proliferative activity of three oxidized ARPs in RAW264.7 mouse macrophages. As shown in Figure 2A, ARP and O-ARP were not cytotoxic to cells at concentrations ranging from 5 to 120 μg/mL, as evidenced by a relative cell survival rate above 90%. O1-ARP consistently outperformed ARP and low-substituted polysaccharides in stimulating macrophages at concentrations ranging from 5 to 120 μg/mL in a dose-dependent manner. Additionally, O1-ARP significantly stimulated the phagocytic activity of macrophages in a dose-dependent manner at concentrations of 5–120 μg/mL, as shown in Figure 2B. However, O3-ARP was weaker than ARP in its stimulatory effect at low concentrations (5–60 μg/mL), though the stimulatory activity of all oxidized samples was better than that of ARP overall. Furthermore, ARP and the three oxidized ARP samples significantly promoted NO production and the secretion of cytokines (IL-6, TNF-α, IL-1β, and NO) at concentrations of 50–200 μg/mL, showing a clear dose–response relationship (Figure 2C–F). Overall, the immunomodulatory effect of oxidized ARP on macrophages in vitro was notable and closely related to the degree of substitution. The higher the degree of substitution, the more effective it was in enhancing cell proliferation, phagocytosis, and cytokine secretion. This phenomenon likely stems from the introduction of hydrophilic carboxyl groups during oxidative modification, which reduces the formation of intra- and intermolecular hydrogen bonds in polysaccharides, thereby enhancing hydroxyl group exposure [25,26].

### 2.4. Formation and Injectability of GE-Os

The *Arnebia euchroma* polysaccharide oxidized by sodium periodate is rich in dialdehyde structures (-CHO), which as active reaction sites can undergo Schiff base reactions with amino groups (-NH_2_) on gelatin to form dynamic Schiff base structures containing carbon–nitrogen double bonds (C=N). This reaction promotes cross-linking between the dialdehyde *A. euchroma* polysaccharide and gelatin molecules, gradually constructing a 3D network structure.

**Figure 3:**
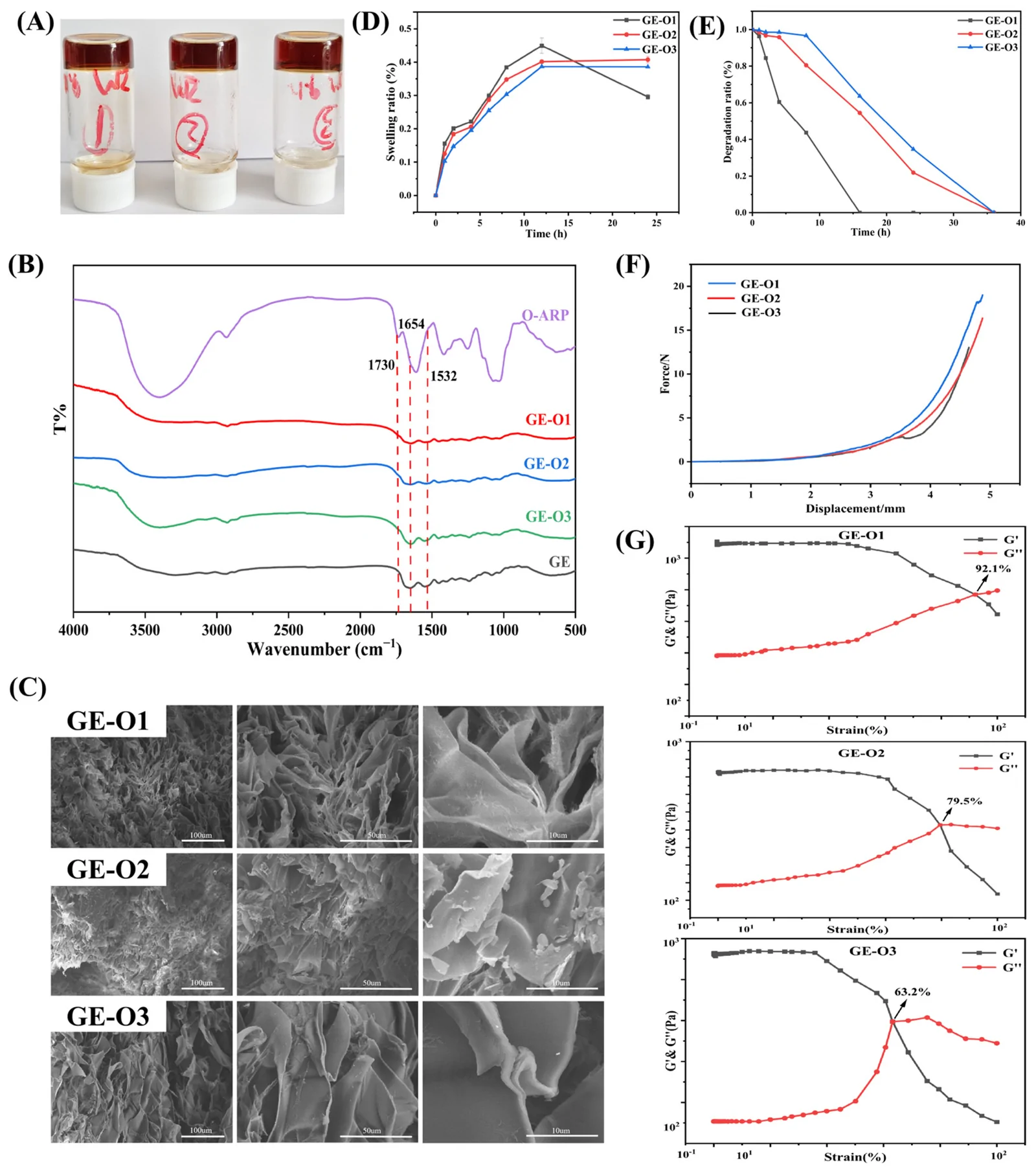
Characterization of the oxidized ARP hydrogel GE-Os. (**A**) Formation of the oxidized ARP hydrogels. (**B**) Fourier transform infrared (FT-IR) spectra of GE-Os. (**C**) Scanning electron microscopy (SEM) images of GE-Os. The SEM images were captured at magnifications of 300×, 1000×, and 5000×, respectively. Left images: 500× magnification; middle images: 1000× magnification; right images: 5000× magnification. (**D**) Swelling curves of GE-Os. (**E**) Degradation curves of GE-Os. (**F**) Mechanical properties of GE-Os. (**G**) Rheological oscillatory strain scan curves of GE-Os.

As shown in Figure 3A, injectable and biodegradable hydrogels named GE-O1, GE-O2, and GE-O3 were constructed using oxidized polysaccharides (O-ARPs) containing aldehyde groups and gelatin with amino groups to induce stable imine bonding. The preformed hydrogels in the syringes were successfully extruded via 22-gauge needles to form shapes, indicating that the hydrogels had good injectability, which is also confirmed by video evidence shown in Supplementary Materials File S1.

### 2.5. Characterization and Properties of GE-Os

#### 2.5.1. FT-IR Analysis

The hydrogels formed by oxidized ARPs and gelatin were characterized by FT-IR. As shown in Figure 3B, compared with unmodified polysaccharide ARP, the characteristic absorption band of the O-H functional group at 3400 cm^−1^ and the C-H band at 2932 cm^−1^ gradually weakened. The vibrational band of the aldehyde group at 1730 cm^−1^ disappeared, along with the stretching vibration absorption peak of the C=O bond at 1654 cm^−1^ (amide I band). Additionally, the absorption peak associated with N-H stretching vibration appeared at 1532 cm^−1^ (amide II band). These observations confirm the successful formation of imine bonds between the aldehyde groups of O-ARP and the amide groups of gelatin.

#### 2.5.2. Scanning Electron Microscopy

The microstructure of the GE-Os, with O-ARPs in different substitution degrees, was comprehensively evaluated by scanning electron microscopy (SEM). Figure 3C presents the microscopic morphology and pore size distribution of the three oxidized polysaccharide hydrogels. As shown in Figure 3D (left), the pore size of GE-O1 was the smallest, and its overall tightly arranged appearance was attributed to the presence of more aldehyde groups in O1-ARP, which formed more imine bonds with GE. This increased the cross-linking density of the Schiff base hydrogel and resulted in a denser network structure. Additionally, physical cross-linking leads to a tighter arrangement of intra- and intermolecular chains, forming more tangles and influencing the porosity of the network [27]. In Figure 3C (middle), the structure of GE-O3 appears relatively looser, with a larger pore size and a fluffy overall appearance. In Figure 3C (right), the structure of GE-O2 is denser compared to that of the GE-O3 gel, with tighter connections between fibrous structures. These results indicate that the microstructure of the three hydrogels became progressively denser with an increase in aldehyde group content. The microstructure of hydrogels is closely related to their mechanical properties and application potential. For example, smaller pore sizes can provide hydrogels with stronger mechanical properties [22], and the porosity and cross-linking density affect their drug delivery and release behaviors. Therefore, hydrogels with different structures and properties can be prepared by selecting various degrees of substitution for aldehyde-based polysaccharides to regulate the cross-linking degree of the hydrogels.

#### 2.5.3. The Degree of Cross-Linking

The cross-linking degrees of the three hydrogels—GE-O1, GE-O2, and GE-O3—were 87.42%, 72.82%, and 52.53%, respectively, as determined using the standard curve from the ninhydrin colorimetric method (Figure S2). The oxidation degree of O-ARP was directly evaluated against the increase in aldehyde groups, and more active sites led to the formation of additional cross-linking sites in the hydrogels, thereby significantly increasing the degree of cross-linking. Zhang et al. prepared oxidized dextran (Odex) with different oxidation degrees using the periodate oxidation method and cross-linked it with chitosan and ε-polylysine. The study showed that the aldehyde groups in Odex covalently cross-linked with amino groups through Schiff base reactions. As the oxidation degree of Odex increased, the degree of covalent cross-linking also increased, resulting in a denser hydrogel structure [28].

#### 2.5.4. Swelling and Degradation Properties

Swelling and degradation properties are essential for evaluating the features of hydrogels and estimating their potential applications in drug delivery systems. Figure 3D illustrates the swelling behavior of the GE-Os in PBS at room temperature. The swelling equilibria of GE-O1, GE-O2, and GE-O3 were reached after 12 h. The swelling rates were 38.65%, 40.16%, and 44.94%, respectively. The swelling curve of GE-O3 exhibited a “rising and then falling” pattern due to its lower cross-linking density and loose network structure, and significant mass loss was observed in GE-O3. Conversely, GE-O1 had a more uniform cross-linking structure or contained stronger hydrophilic groups, allowing it to maintain stable swelling. These two phenomena are closely related to the cross-linking capacity of the hydrogel. An increase in aldehyde group content enhances the cross-linking property, leading to a denser hydrogel structure. This densification constrains the diffusion of solvent molecules throughout the hydrogel. Stronger interactions between polymer chains also require more space and time for chain extension, which results in a slower dissolution rate of the hydrogel [29].

The hydrogels were placed at swelling equilibrium in an incubator at 37 °C, and their weight changes were continuously observed. The degradation curves are shown in Figure 3E. Due to its lowest cross-linking capacity and unstable structure, GE-O3 degraded the earliest and was completely degraded within 16 h. GE-O2 and GE-O1 maintained relatively stable structures within the first 8 h and began to degrade thereafter. The degradation rate of GE-O1 was slightly slower than that of GE-O2, and both were fully degraded after 36 h [30]. Swelling and degradation behaviors are crucial for assessing a hydrogel’s suitability for wound healing and drug delivery applications. Therefore, ideal drug delivery materials require a delicate balance between swelling capacity and structural stability. The current results are consistent with recent reports indicating that oxidized polysaccharide hydrogels possess favorable physical and chemical properties [15,31].

#### 2.5.5. Mechanical Properties

Figure 3F depicts the force–displacement relationship for the GE-Os. Analysis of the hysteresis curves revealed that, under identical displacement conditions, the force for GE-O1 increased most rapidly and reached the highest final magnitude. This indicates that GE-O1 possesses the greatest resistance to deformation and the highest mechanical strength. In contrast, GE-O2 exhibited intermediate force values, while GE-O3 consistently displayed the lowest, signifying its weaker mechanical strength and relatively loose cross-linking network structure. Notably, at displacements of 4–5 mm, the force generated by GE-O1 was significantly higher than that of GE-O2 and GE-O3, indicating its superior mechanical load-bearing capacity. The enhanced mechanical strength of the GE-O hydrogels is attributed to the presence of aldehyde groups, which reinforce the internal stability of the Schiff base networks. Hydrogels with strong mechanical properties are well suited, as durable matrices, for sustained drug release. Their stable network structures support controlled release kinetics, making them particularly advantageous for applications requiring long-term localized delivery, such as anticancer therapy or chronic inflammation management. For instance, Sun et al. reported an injectable, self-adaptive hydrogel delivery system with robust mechanical properties for encapsulating the antifibrotic drug rapamycin. This hydrogel achieved controlled and sustained release, effectively inhibiting subconjunctival fibrosis [32].

#### 2.5.6. Rheology Analysis

The viscoelasticity of GE-Os was compared using the crosspoint of the energy storage modulus (G′) and loss modulus (G″) in strain amplitude sweep tests. As shown in Figure 3G, different strains (0.1–100%) were applied while keeping the angle, frequency, and temperature constant. All three hydrogels maintained their internal structure without collapsing under stresses of up to 10%. Among them, GE-O1 showed the intersection of G′ and G″ at a strain of 92.1%, while the collapse points of GE-O2 and GE-O3 were 79.5% and 63.2%, respectively. This indicates that hydrogel cross-linking gradually tightens and the collapse point shifts backward with increasing aldehyde group content. The extension of cross-linking strength thereby enhances the mechanical properties of hydrogels [11].

### 2.6. Molecular Dynamics Simulation of GE-Os

**Figure 4:**
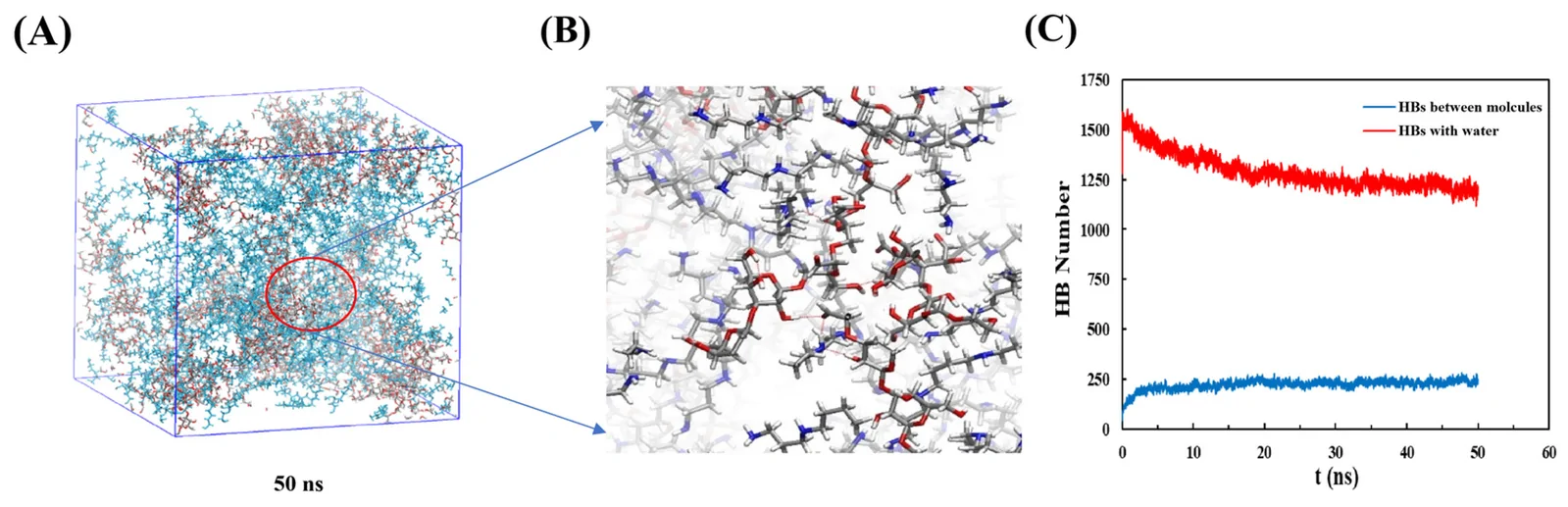
Molecular dynamics (MD) simulation mechanism of GE-Os. (**A**) Structural changes in the hydrogel at 50 ns. (**B**) Self-assembled structure and intermolecular interactions of the oxidized ARP hydrogel at a stable state (50 ns). (**C**) Dynamic changes in the number of hydrogen bonds between molecules and with water.

The spatial arrangement of polysaccharide hydrogel chains, the distribution of cross-linking points, and the movement of chain segments were simulated using MD to predict the 3D network formation mechanism of the hydrogels. Figure 4A illustrates the MD self-assembly process of oxidized arabinogalactan (1:3) and gelatin molecules with short amine chains over 0–50 ns. At the beginning of the simulation (0 ns), the dialdehyde polysaccharides (red) and gelatin (blue) displayed a random distribution within the simulation box. The molecules were relatively distant from one another and had few interactions. After 1 ns of simulation, the molecules began to aggregate, and locally, the red and blue molecules moved closer together, indicating the onset of intermolecular interactions. After 50 ns of simulation, the gel structure became stable, and intermolecular interactions reached equilibrium, with the gel network uniformly distributed in 3D space, as shown in Figure 4B. This stabilized structure strongly influences the physical and chemical properties of the hydrogels. Hydrogen bonding serves as a key parameter for visualizing dynamic connectivity and water interactions. The dynamic rheological behavior of polymer aqueous solutions is predominantly governed by intermolecular hydrogen bonds, which play a critical role in network structure formation [33]. Changes in the number of hydrogen bonds (HBs) between molecules and water are illustrated in Figure 4C. With increasing simulation time, hydrogen bonding with water molecules gradually decreased, clustering of molecules occurred, and hydrogen bonding between molecules rapidly increased until equilibrium was reached.

Through the binding energy profile over the duration of the simulation, it was found that the energy gradually converged in the NPT modeling process at 298 K, indicating that the system reached equilibrium (Figure S3A). The binding energy of intermolecular interactions between molecules decreased rapidly and reached equilibrium (Figure S3B), while the interaction energy with water molecules gradually increased. The more negative the interaction energy, the stronger the force of interaction. Energy shifts in Coulomb and van der Waals (VDW) forces showed that the energy decrease in VDW forces was significantly greater than that of Coulomb forces in intermolecular interactions, whereas the energy increase in VDW forces was markedly higher than that of Coulomb forces in water molecules. Notably, binding energy analysis indicated that van der Waals forces between oxidized polysaccharides and gelatin were the main driving force of self-assembly throughout the process. Furthermore, the solvent-accessible surface area (SASA) value was a pivotal factor affecting the stability and compactness of the molecular system [34]. The SASA of the hydrogel system increased rapidly at the beginning of the simulation and then decreased sharply after 20 ns (Figure S3C), indicating that the system compacted over the simulation time.

The molecular dynamics results demonstrated that oxidized ARPs and gelatin may form a stable 3D network structure through hydrogen bonding and van der Waals forces. Combined with infrared spectroscopy results, it is likely that hydrogen bonding, maintaining structural stability, also occurs near the imine bonds (C=N) generated through the Schiff base reactions. For example, in protein structures, after certain amino acid residues form covalent bonds through Schiff base reactions, the surrounding residues may further stabilize the structure through hydrogen bonding [35].

### 2.7. In Vitro Biocompatibility and Immunomodulatory Activity of GE-Os

**Figure 5:**
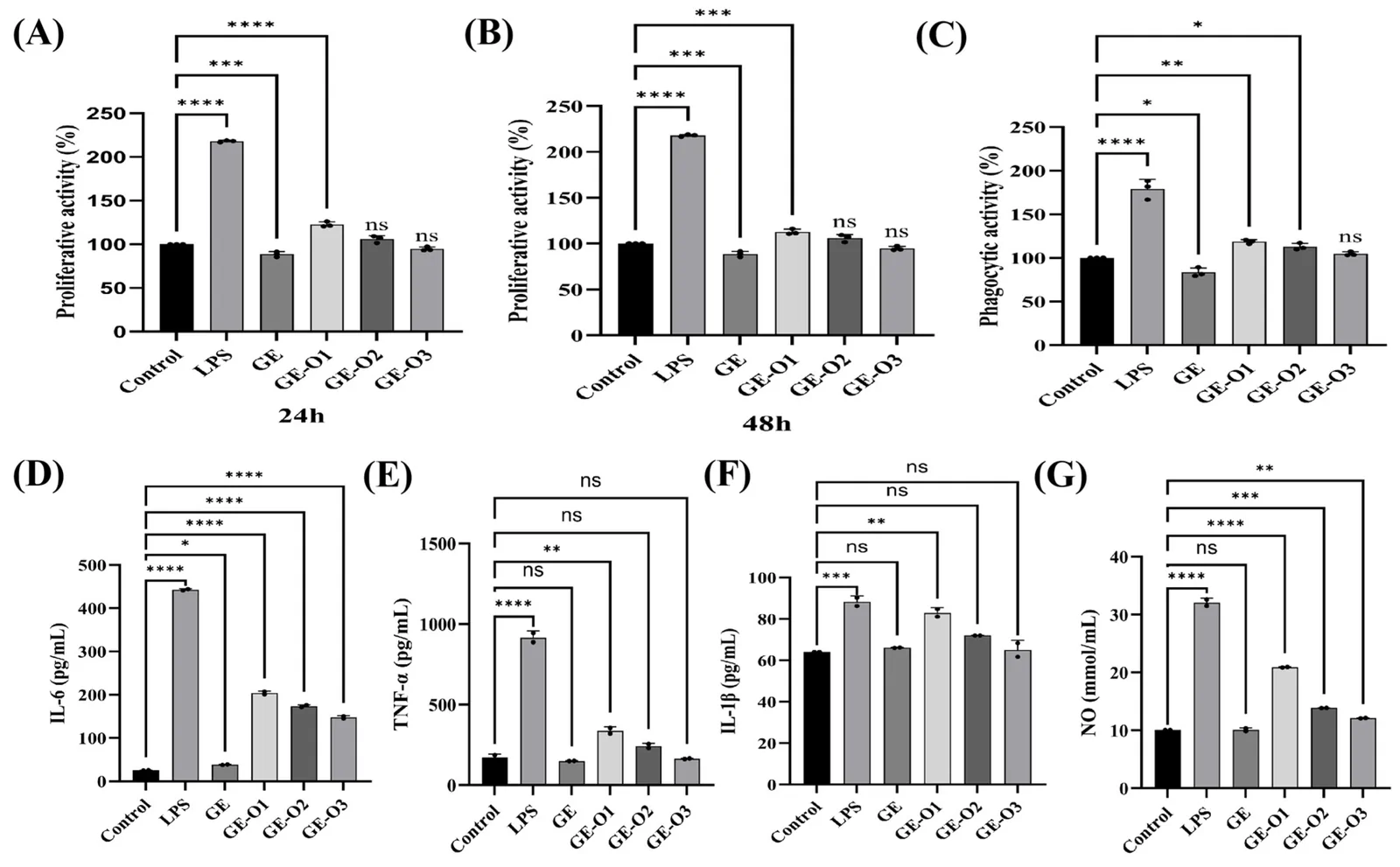
Immunomodulatory activity of the extracts of the three oxidized ARP hydrogels on RAW264.7 cells. RAW264.7 cells were cultured in the presence of different samples for 24 h. Macrophage proliferation activity at 24 h (**A**). Macrophage proliferation activity at 48 h (**B**). Phagocytic activity (**C**). Culture supernatant was collected and assayed for the levels of IL-6 (**D**), TNF-α (**E**), IL-1β (**F**) and NO (**G**). LPS was used as a positive control. * *p* < 0.05, ** *p* < 0.01, *** *p* < 0.001 and **** *p* < 0.0001 versus cell control; ns indicates no significant difference (*p* ≥ 0.05). Results are expressed as mean ± SD (*n* = 3).

The cytocompatibility and cytotoxicity of GE-Os were investigated by co-culturing hydrogel extracts with macrophages for 24 h and 48 h. As shown in Figure 5A,B, GE-O1, GE-O2, and GE-O3 exhibited no obvious cytotoxicity (cell viability > 90%) and significantly promoted macrophage proliferation. Their stimulatory activity was consistently higher than that of GE alone and lower than that of the positive control LPS group, suggesting the well-established biocompatibility of these materials. Compared with the negative control group, the proliferative activity of macrophages stimulated by GE-O1 was notably higher, and proliferation after 24 h of co-culture was superior to that after 48 h. Subsequent experiments on phagocytosis, NO production, and cytokine secretion (TNF-α, IL-1β, and IL-6) were conducted following 24 h of co-culture.

As displayed in Figure 5C, the phagocytic activity of the three oxidized polysaccharide hydrogel groups (GE-O1, GE-O2, and GE-O3) in macrophages was consistently higher than that of the gelatin group (GE) and lower than that of the positive control group (LPS). Compared with the negative control, GE-O1 (** *p* < 0.01) and GE-O2 (* *p* < 0.05) showed significant differences, while GE-O3 showed no significant difference (ns).

The immunostimulatory activity of GE-O1, GE-O2, and GE-O3 was further evaluated by measuring cytokine levels and NO production in RAW264.7 cells. As shown in Figure 5, GE induced only slight cytokine production, whereas GE-O1, GE-O2, and GE-O3 induced higher cytokine levels, especially GE-1, which stimulated about 20-fold higher IL-6 and 10-fold higher NO compared with the control (Figure 5D,G). The TNF-α and IL-1β results (Figure 5E,F) showed that the GE-O1 group was significantly different from the blank group (** *p* < 0.01), while GE-O2 and GE-O3 showed no significant differences. Overall, GE-O1 exhibited strong immunomodulatory activity, especially in stimulating macrophages to secrete IL-6 and NO. These findings provide a cellular-level experimental basis for evaluating the inflammation-regulating properties and potential biocompatibility of hydrogel materials. However, the observed alterations in macrophage proliferation, phagocytosis, and cytokine secretion upon exposure to hydrogel extracts raise a pragmatic concern regarding potential acute local toxicity at the injection site, which warrants further in vivo investigation.

### 2.8. Formation of CpG ODN–Loaded Hydrogel Complexes (GE-Os-CpG)

#### 2.8.1. Zeta Potential of GE-Os-CpG

Electrostatic attraction and repulsion play vital roles in drug carriers within hydrogels. The zeta potentials of the oxidized hydrogels (GE-Os) and the hydrogel complexes (GE-Os-CpG) were measured, and the results are presented as mean ± SD (*n* = 3) in Figure S4. As shown in Figure S4, the zeta potentials of GE-O1, GE-O2, and GE-O3 were 53.52 ± 2.18 mV, 43.79 ± 3.05 mV, and 33.20 ± 2.67 mV, respectively, which were significantly higher than that of unmodified GE (15.2 ± 2.1 mV, *p* < 0.05). The positive charges are attributable to the abundant aldehyde groups introduced by periodate oxidation. After complexation with CpG ODNs (1862), the zeta potentials of the three hydrogel complexes (GE-O1-CpG, GE-O2-CpG, and GE-O3-CpG) decreased markedly to 27.33 ± 2.54 mV, 26.55 ± 3.12 mV, and 12.93 ± 2.08 mV, respectively, with all showing statistically significant differences compared to their corresponding GE-O precursors (*p* < 0.05). This decrease confirms that electrostatic interactions occurred between the positively charged hydrogel network and the negatively charged CpG ODNs. Importantly, all three GE-O-CpG complexes retained a net positive surface charge, which would facilitate their cellular uptake via electrostatic attraction to negatively charged cell membranes.

#### 2.8.2. Release Performance of GE-O1-CpG Complex

**Figure 6:**
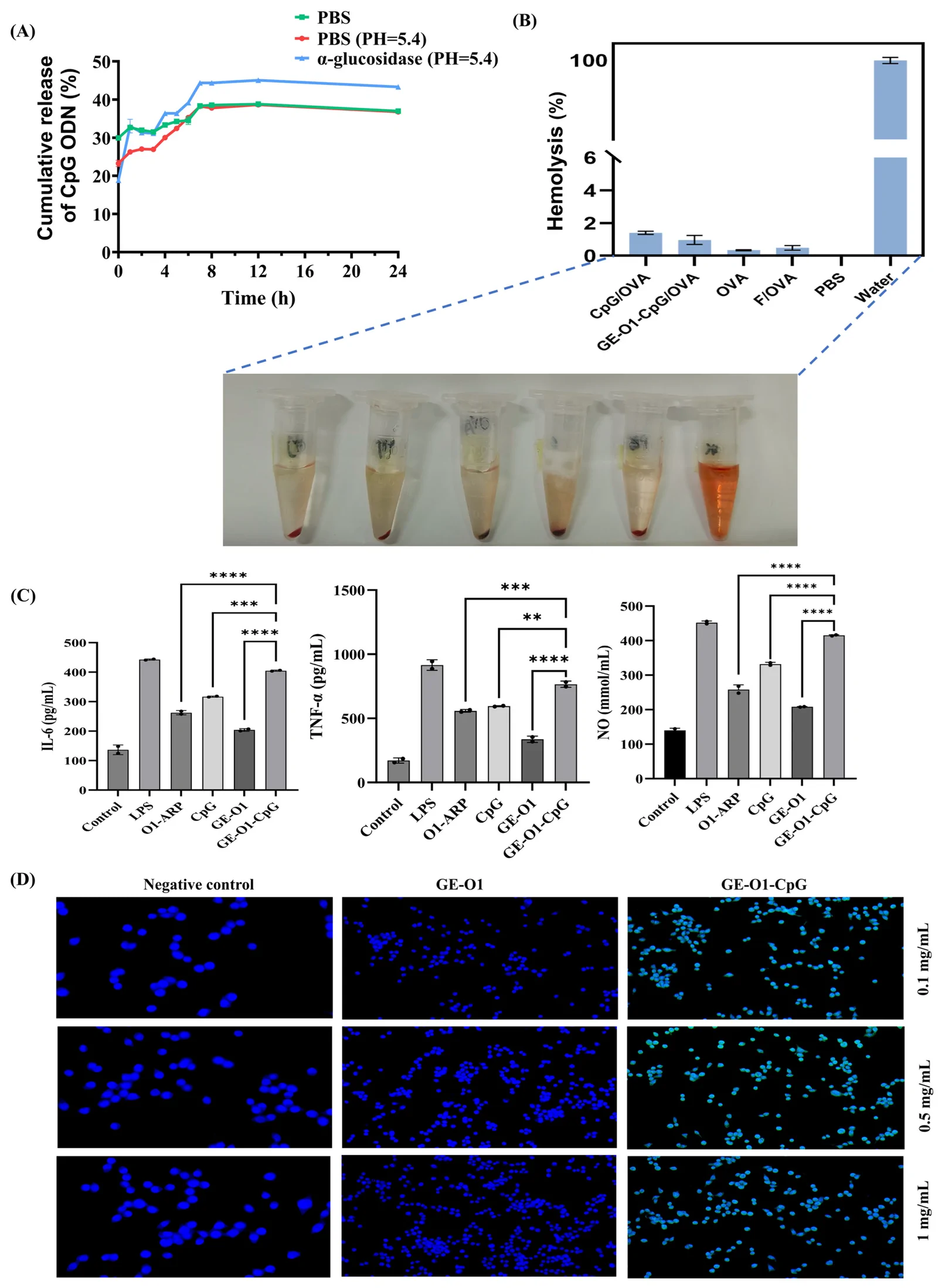
(**A**) In vitro release profile of CpG ODNs from GE-O1-CpG hydrogel complex under different environments. (**B**) Hemolysis ratio of GE-O1-CpG hydrogel complex. (**C**) Immunomodulatory activities of GE-O1 hydrogel and GE-O1-CpG: IL-6, TNF-α, and NO production. (**D**) Cellular uptake of fresh culture medium, GE-O1 without FAM-CpG ODN 1862, and drug-loaded hydrogel complex with FAM-CpG ODN 1862 in RAW264.7 macrophages stained with DAPI. Results expressed as mean ± SD (*n* = 3). ** *p* < 0.01, *** *p* < 0.001 and **** *p* < 0.0001.

The loading capacity of GE-O1 was 63.4%, as calculated in Supplementary Materials File S2. Figure 6A shows the cumulative release profiles of CpG ODNs from the hydrogel complex under three environments: physiological PBS, acidic PBS (pH = 5.4; tumor microenvironment), and acidic PBS containing α-glucosidase (pH = 5.4). All groups exhibited an initial slow-release phase (0–6 h), followed by divergent kinetics. In the presence of α-glucosidase at pH 5.4, the cumulative relative release of CpG ODNs increased rapidly from approximately 18.80% at the initial time point to a peak of about 45.04% within the first 8 h, and then gradually plateaued at roughly 43.30% over the remaining period up to 24 h. In contrast, the acidic PBS (pH 5.4) and PBS groups exhibited relatively slower and lower release behaviors, with cumulative release percentages reaching approximately 38.63% and 38.79% at 8 h, respectively, and maintaining stable plateau levels of around 37% at 24 h. The significantly accelerated initial release in the α-glucosidase (pH 5.4) group indicates that the acidic and enzymatic environment effectively promoted a faster and higher cumulative release of CpG ODNs compared with the PBS controls. These findings demonstrate that environmental triggers (pH + α-glucosidase) might enable spatiotemporally controlled drug delivery. Enzyme-responsive degradation optimizes therapeutic payload release at target sites, achieving maximal release efficiency in pathologically relevant microenvironments. Zhang et al. constructed NH_2_-Gly/CpG drug-loaded complexes for nucleic acid delivery. In vitro release studies showed limited CpG ODN release (20%) in PBS (pH 5.4). In contrast, α-glucosidase significantly accelerated CpG ODN release from the NH_2_-Gly/CpG complex, demonstrating that α-glucosidase-mediated hydrolysis rapidly decomposed the complex, resulting in efficient payload release [36].

#### 2.8.3. Hemocompatibility

Before biological application, hemocompatibility assessment is essential. The hemolysis assay is a critical method for evaluating the blood compatibility of biomaterials. Hemolysis testing was performed using PBS and ultrapure water as negative and positive controls, respectively. As demonstrated in Figure 6B, OVA and the different adjuvant groups, upon direct contact with erythrocytes (red blood cells), did not induce rupture or dissolution, indicating favorable hemocompatibility. Visual inspection revealed that the supernatant in the GE-O1-CpG/OVA group was clear and transparent, comparable to the PBS control group, confirming the absence of hemolysis. In contrast, significant hemolysis was observed in the water control group. Quantitative analysis showed that the hemolysis rate of all sample groups was <5% (Figure 6B), thereby meeting the international standard for hemocompatible materials. Collectively, these findings demonstrate the excellent blood compatibility of the hydrogel complex adjuvant [36].

#### 2.8.4. In Vitro Immunomodulatory Activity of GE-O1-CpG

As shown in Figure 6C, the immunomodulatory activity of the drug-loaded hydrogel complex (GE-O1-CpG) was evaluated by measuring the release of inflammatory factors IL-6, TNF-α, and NO. Based on the secretion levels of these cytokines, the stimulatory activity of GE-O1-CpG on macrophages was significantly higher than that of the GE-O1 and CpG ODN 1826 groups, suggesting that GE-O1-CpG is an effective hydrogel for CpG ODNs with synergistic immunostimulatory effects.

#### 2.8.5. Cellular Uptake Assay of GE-O1-CpG

The cellular uptake assay is a key prerequisite for evaluating the delivery efficiency and biosafety of drug-loaded carriers in the body. As shown in Figure 6D, the negative control group and the black group displayed only blue nuclear fluorescence with DAPI staining and no green fluorescence signal, indicating no non-specific uptake of FAM-CpG ODN 1862 by cells without the hydrogel complex. This excluded interference from cellular autofluorescence or external contamination. Additionally, the number of cells in the black group increased significantly compared with the negative control group containing only medium, suggesting that the oxidized polysaccharide hydrogel promoted cell proliferation. These results were consistent with the in vitro cell viability results, further confirming that the hydrogel carrier exhibited potential biosafety in the cellular level.

In the experimental groups, green fluorescence was markedly enhanced at 0.1–1 mg/mL concentrations of GE-O1 hydrogel complexes. These findings indicate that the GE-O1 hydrogel can load FAM-CpG ODN 1862 by electrostatic adsorption and effectively mediate its uptake by RAW264.7 cells. This successfully verified the effectiveness of GE-O1 as a nucleic acid delivery system and provided an experimental basis for further investigating the immune regulation mechanism of CpG ODN 1862, such as activation of intracellular immune signaling pathways at the delivery level.

### 2.9. Therapeutic Evaluation of GE-O1-CpG/OVA Adjuvant In Vivo

#### 2.9.1. Biosafety Analysis

**Figure 7:**
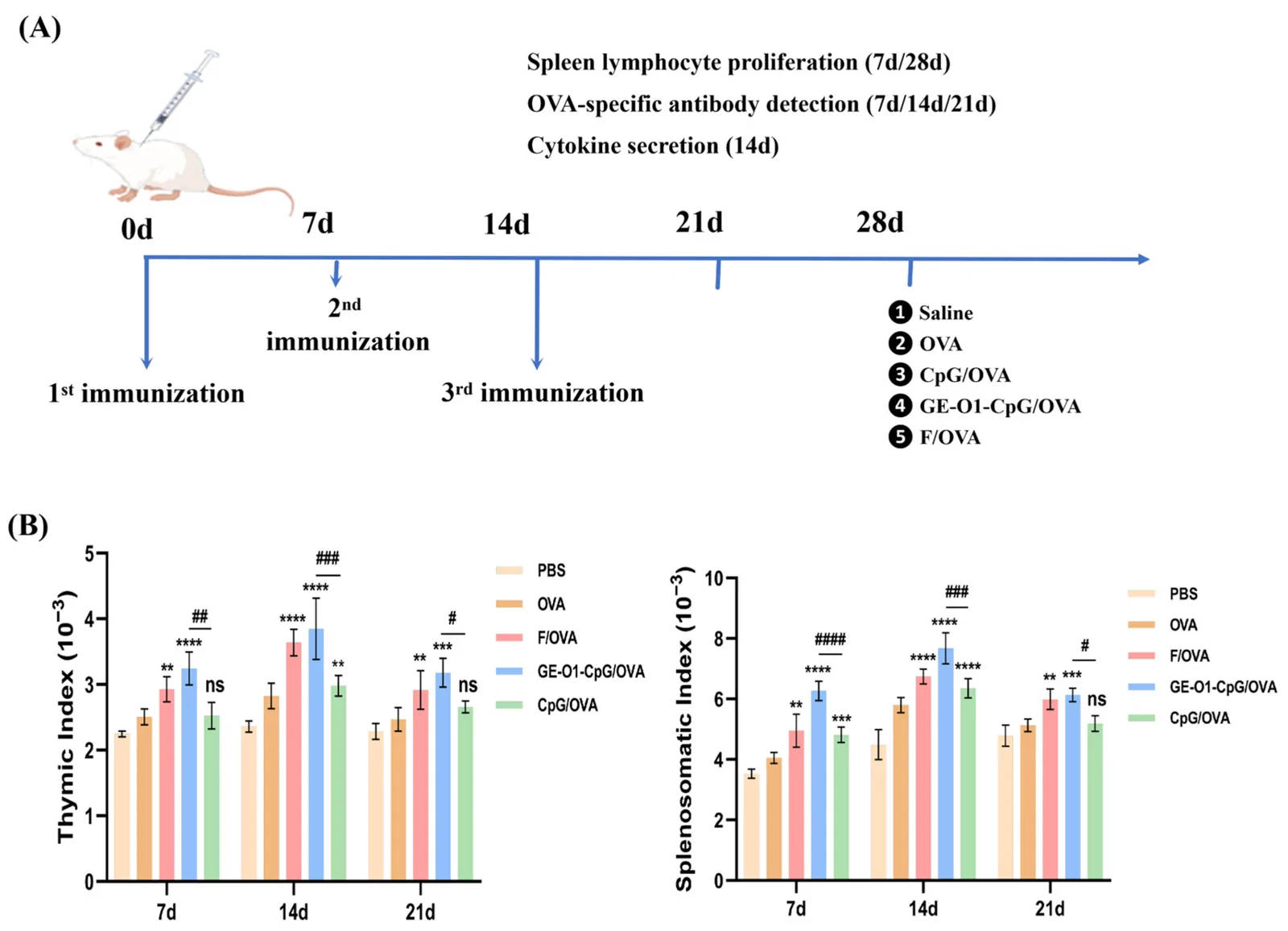
Safety assay of the GE-O1-CpG hydrogel complex in vivo. (**A**) Immunization procedures for mice. (**B**) Determination of immune organ indexes measured on days 7, 14, and 21. All results presented as mean ± SD (*n* = 3). ** *p* < 0.01, *** *p* < 0.001 and **** *p* < 0.0001 versus OVA; ns indicates no significant difference (*p* ≥ 0.05); # *p* < 0.05, ## *p* < 0.01, ### *p* < 0.001 and #### *p* < 0.0001 for GE-O1-CpG/OVA versus CpG/OVA.

Owing to its excellent biocompatibility and strong immunostimulatory properties, the in vivo therapeutic effects of the hydrogel complexes were further evaluated using ICR mice. To further investigate the in vivo immunoenhancing effects, we established a prime–boost immunization animal model (Figure 7A). The in vivo toxicity of the constructed GE-O1-CpG/OVA was assessed by monitoring animal body weight (Figure S5), histopathological examination, organ indices (Figure S6) and biochemical and hematological analyses. No significant differences in serum functional parameters (AKP, ALT, AST, BUN, and LDH) were observed between the GE-O1-CpG/OVA group and the control group, indicating no apparent toxicity to the liver or kidneys in Figure S7. To evaluate the systemic immune response elicited by immunization, the spleen index and thymus index of mice were measured on days 7, 14, and 21 post-immunization. On day 7, immunization induced a significant and progressive increase in both indices compared with control levels in the F/OVA (*p* < 0.01), GE-O1-CpG/OVA (*p* < 0.0001), and CpG/OVA (*p* < 0.001, ns) groups (Figure 7B). The spleen and thymus indices of GE-O1-CpG/OVA peaked at day 14 post-immunization, reaching their highest values and significantly exceeding those of CpG/OVA (*p* < 0.001).

By day 21, the spleen and thymus indices of all groups showed a marked decrease from their peaks, while the F/OVA (*p* < 0.01) and GE-O1-CpG/OVA (*p* < 0.001) groups remained at relatively high levels, suggesting a partial resolution of the splenomegaly associated with the acute immune response phase. These dynamic changes in organ indices reflect the temporal progression of the systemic immune response: early activation and lymphocyte proliferation/recruitment occurred mainly in the spleen and thymus, followed by a resolution phase. H&E-stained pathological examination of multiple organs confirmed that no persistent organ damage was detectable 28 days post-primary vaccination (Figure S8). Nevertheless, the current dataset does not indicate the definitive biocompatibility of the hydrogel; instead, it underscores the critical steps for dose- and time-dependent studies to establish a safety window and evaluate local inflammatory dynamics.

#### 2.9.2. GE-O1-CpG/OVA as an Adjuvant Enhanced Humoral and Cellular Immunity

**Figure 8:**
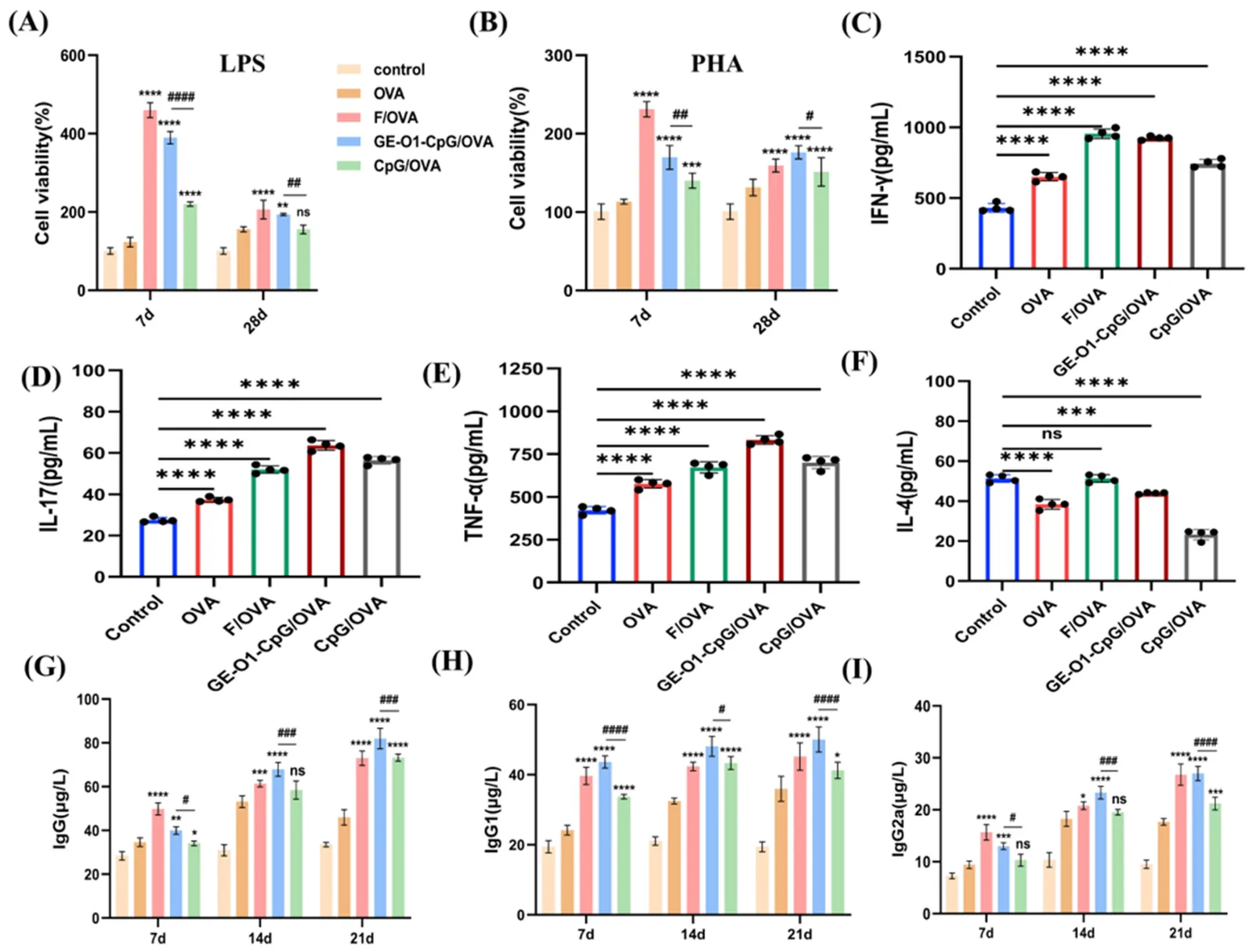
GE-O1-CpG/OVA as an immunological adjuvant enhancing humoral and cellular immunity in vivo. Splenocyte proliferation of the GE-O1-CpG hydrogel complex in vivo at 7 and 21 days post-immunization (*n* = 3). Effects on splenic lymphocyte proliferation in synergistical stimulation with LPS (**A**); effects on splenic lymphocyte proliferation in synergistical stimulation with PHA (**B**). Cytokine secretion in splenocyte supernatants at 14 days post-immunization (*n* = 4): IFN-γ level (**C**), IL-17 level (**D**), TNF-α level (**E**), and IL-4 level (**F**). Serum expressions of OVA-specific IgG (**G**), isotypes of IgG, IgG1 (**H**) and IgG2a (**I**) measured on day 7, 14, and 21 by ELISA kits (*n* = 3). All results presented as mean ± SD. * *p* < 0.05, ** *p* < 0.01, *** *p* < 0.001 and **** *p* < 0.0001 versus OVA; ns indicates no significant difference (*p* ≥ 0.05); # *p* < 0.05, ## *p* < 0.01, ### *p* < 0.001 and #### *p* < 0.0001 for GE-O1-CpG/OVA versus CpG/OVA.

The immunoadjuvant potential of GE-O1-CpG/OVA was evaluated by measuring its ability to stimulate splenocyte proliferation in vivo. As shown in Figure 8A,B, splenocyte proliferation was assessed via a cell viability assay on days 7 and 28 post-primary-immunization. On day 7, the GE-O1-CpG/OVA group exhibited significantly higher proliferation rates than all groups except F/OVA (*p* < 0.05). Under combined LPS and PHA stimulation, GE-O1-CpG/OVA induced the strongest proliferative response among all treatments, with highly significant differences versus CpG/OVA (*p* < 0.0001 or *p* < 0.01). On day 28, proliferation rates declined across all groups; however, GE-O1-CpG/OVA and F/OVA maintained significantly higher activity compared to other controls (*p* < 0.05). Notably, with PHA costimulation, GE-O1-CpG/OVA surpassed even F/OVA while retaining significant superiority over CpG/OVA (*p* < 0.05) in Figure 8B. Collectively, these results demonstrate that GE-O1-CpG/OVA exhibits potent immunostimulatory capacity in promoting splenocyte proliferation upon antigen restimulation, outperforming conventional adjuvants in sustaining this response. As depicted in Figure 8C–E, the OVA, F/OVA, CpG/OVA, and GE-O1-CpG/OVA groups exhibited significantly elevated secretion of IFN-γ, IL-17, and TNF-α compared with the control group (*p* < 0.0001). The levels of the Th1 cytokine IFN-γ in the GE-O1-CpG/OVA group were not significantly different from those in the F/OVA group but were higher than those in the OVA and CpG/OVA groups, as shown in Figure 8C. Similarly, the secretion levels of IL-17, the Th17-associated cytokine, and TNF-α in the GE-O1-CpG/OVA group were significantly higher than in the other groups. In addition, the Th2-associated cytokine IL-4 was quantified in serum samples post-immunization. Compared with the control group, GE-O1-CpG/OVA induced significantly higher IL-4 secretion on day 14 (*p* < 0.00001). Importantly, GE-O1-CpG/OVA outperformed CpG/OVA, producing 1.4-fold higher IL-4 levels (*p* < 0.0001), as shown in Figure 8F. Although many adjuvants induce strong antibody responses, most stimulate weak immune responses in Th1 and especially Th17 cells [37]. GE-O1-CpG may trigger key cytokines (IL-17, IFN-γ, etc.) to induce persistent and long-lasting immune responses in vivo.

The efficacy of a vaccine is critically dependent on the magnitude, quality, and persistence of the antigen-specific humoral immune response it elicits [38]. The measurement of total IgG provides a global indicator of the overall strength of the humoral response induced by a vaccine formulation. Particularly, the subclass IgG1 is predominantly associated with a Th2-type immune response, characterized by the production of cytokines such as IL-4 and IL-5. Conversely, the IgG2a isotype is a hallmark of a Th1-type immune response, driven by cytokines such as IFN-γ and IL-12 [39]. The dynamic changes in OVA-specific IgG antibody levels were assessed by enzyme-linked immunosorbent assay (ELISA) on days 7, 14, and 21. As shown in Figure 8G, on day 7 post-primary-immunization, serum IgG antibody levels in the GE-O1-CpG/OVA group were slightly lower than those in the F/OVA group but significantly higher than those in the OVA and CpG/OVA groups (*p* < 0.05). From day 14 to 21, the GE-O1-CpG/OVA group exhibited significantly higher serum IgG levels compared to all other groups (control, OVA, F/OVA, and CpG/OVA). By day 21, the GE-O1-CpG/OVA group showed the highest IgG levels among all groups, with a particularly significant difference compared to the CpG/OVA group (*p* < 0.001). As shown in Figure 8H,I, levels of IgG subclasses (IgG1 and IgG2a) in the GE-O1-CpG/OVA group gradually increased from day 7 to day 21 post-immunization, peaking after day 21 and thereby accelerating initiation and amplifying the magnitude of the humoral immune response. Concurrently, both F/OVA and CpG/OVA adjuvants also induced secretion of IgG, IgG1, and IgG2a antibodies. These results demonstrate that, in contrast to Freund’s adjuvant and the nucleic acid-only adjuvant (CpG/OVA), GE-O1-CpG/OVA elicits a potent and sustained immune response. Collectively, these findings indicate that GE-O1-CpG/OVA effectively enhances antigen-induced antibody production and promotes sustained humoral immunity.

## 3. Conclusions

This study successfully developed an injectable immunostimulatory hydrogel based on aldehyde-modified A. euchroma polysaccharide (O-ARP) and gelatin (GE) which intrinsically activates immune cells and enables CpG oligonucleotide delivery. The oxidation-degree-dependent cross-linking mechanism affords programmable immunostimulatory potency, and the GE–O1–CpG complex achieves dual immunological synergy, driving robust humoral and cellular immunity that surpasses conventional adjuvants. However, a critical limitation must be acknowledged: the Schiff base conjugation with gelatin substantially reduced the immunostimulatory activity of O-ARP, likely due to aldehyde group occupancy and poor extractability from the hydrogel matrix. This concern is reinforced by parallel findings that cubosomes loaded with ARP [40] preserved superior activity, suggesting that gelatin hydrogels may not be the optimal vehicle for ARP bioactivity. Consequently, the current formulation entails a trade-off between safety and efficacy, warranting further mechanistic and formulation-focused investigations. Moreover, a limitation of this study is that all experiments for each formulation were performed on a single batch; therefore, the batch-to-batch consistency of the observed physicochemical properties and biological activities requires further investigation. Another major limitation of the current study is that the functional biological assays (e.g., ELISA and phagocytosis) were performed on total populations without normalization to absolute cell numbers, and the present data cannot distinguish between increased activity per cell and increased total cell mass. Therefore, the conclusion that treatment with the GE–O1–CpG complex ‘enhances macrophage function’ should be interpreted with caution and requires confirmation at the single-cell level or via sorted cell assays in future studies. Despite these limitations, our approach offers a rational design strategy for bridging innate and adaptive immunity, though careful evaluation of local inflammatory risks and carrier optimization are prerequisites for clinical translation.

## 4. Materials and Methods

### 4.1. Materials

*Arnebia euchroma* (Royle) Johnst. roots (No. 2312001) were obtained from Xinjiang, China. Gelatin (type B, (C_102_H_151_O_39_N_31_)_n_) and ovalbumin (OVA, ≥98%) were purchased from Sigma-Aldrich, Burlington, MA, USA. Sodium periodate and the nucleic acid drug ODN 1862 (CAS No.: 202668-42-6) were obtained from Macklin Biochemical Co., Shanghai, China. RAW264.7 mouse macrophages were purchased from the Shanghai Cell Bank, Chinese Academy of Sciences (Cat. No. SCSP-5036), Beijing, China. DMEM (Cat. No. 11965092), FBS (Cat. No. 10099141C), and penicillin–streptomycin were purchased from Gibco, Grand Island, NY, USA. Lipopolysaccharide (LPS; Cat. No. L4391) was purchased from Sigma-Aldrich, Taufkirchen, Germany. Mouse IL-1β, TNF-α, and IL-6 precoated ELISA kits were obtained from Shenzhen DaKeWei Biotechnology Co., Ltd. (Batch Nos.: 1210122, 1217202, 1210602), Shenzhen, China. Analytical-grade glycol, ninhydrin, SnCl_2_·2H_2_O, NaOH and Freund’s adjuvant (F#: 1001131105) were supplied by Sinopharm Chemical Reagent Co., Ltd., Shanghai, China. Aspartate aminotransferase (AST), alanine aminotransferase (ALT), alkaline phosphatase (ALP), lactate dehydrogenase (LDH; cardiac function marker), and blood urea nitrogen (BUN; renal function marker) assay kits were purchased from Nanjing Jiancheng Bioengineering Institute, Nanjing, China. Mouse OVA-specific IgG, IgG1, and IgG2a enzyme-linked immunosorbent assay (ELISA) kits were obtained from Aolaibo Technology Co., Ltd., Beijing, China.

### 4.2. Preparation of ARP

ARP was extracted and purified as described in our previous study [18]. Briefly, crude polysaccharides were obtained via hot water extraction, ethanol precipitation, and deproteinization with 5% TCA. The molecular weight was calculated as 1.209 × 10^4^ Da and the polydispersity coefficient of ARP was 1.023, suggesting that ARP is highly uniform, as determined by HPGPC using dextran standards of varying molecular weights. The total sugar content of ARP reached 91%.

### 4.3. Synthesis of Oxidized ARP

Sodium periodate oxidation is a widely used method for polysaccharide modification, offering mild reaction conditions and high selectivity [41]. Dialdehyde ARP was synthesized as previously reported with minor modifications. Briefly, 0.1 g of ARP was dissolved by stirring at 30 °C for 1 h, and the pH was adjusted to 6.6 under varying conditions of sodium metaperiodate dosage, temperature, and reaction time. The reaction was performed in the dark and terminated by adding 5 mL of ethylene glycol, followed by stirring for 30 min. The mixture was dialyzed for 72 h, then freeze-dried at −80 °C and 0.200 mBar for 72 h. The optimal parameters for O1-ARP were determined via single-factor experiments to be 30 °C, an NaIO_4_:ARP ratio of 0.6:1, 8 h, and a pH of 6.6. Concurrently, two additional derivatives were selected for subsequent studies based on their degree of substitution—O3-ARP (lowest) and O2-ARP (moderate)—which were obtained at 60 °C/0.2:1/4 h/pH 6.6 and 25 °C/0.4:1/6 h/pH 6.6, respectively.

#### 4.3.1. Determination of the Degree of Oxidation

The hydroxylamine hydrochloride method [42] was used to quantify HCl released from the reaction between O-ARP and hydroxylamine hydrochloride via acid–base titration. Briefly, 0.1 mg of O-ARP was stirred with 20 mL of hydroxylamine hydrochloride–methyl orange solution at 25 °C for 2 h. The aldehyde concentration of oxidized ARPs (-CHO) was calculated using Equation (1):

ΔV × 0.001 × C_NaOH_ = n_CHO_; [-CHO] = n_CHO_/W (1)

(where ΔV is the volume of NaOH; n_NaOH_ is the molarity of the NaOH standard liquid; n_CHO_ is the molarity of aldehyde groups on the oxidized polysaccharide; W is the mass of the oxidized polysaccharide; and C_NaOH_ = 0.9405 M).

#### 4.3.2. Physicochemical Properties of Oxidized ARP

The molecular weight, monosaccharide composition, and total polysaccharide content of O-ARP were determined using high-performance gel permeation chromatography (HPGPC), ion chromatography, and the phenol–sulfuric acid method, respectively, as described in Supplementary Materials File S3.

### 4.4. Hydrogel Preparation

One gram of gelatin was dissolved in 10 mL of deionized water, and 300 mg of oxidized samples (O1-ARP, O2-ARP, and O3-ARP) were dissolved in 5 mL of deionized water and adjusted to pH 10.0. The mass ratio of gelatin to oxidized polysaccharide was thus 5:3. The O1-ARP, O2-ARP, and O3-ARP solutions were added dropwise to the gelatin (GE) solution with gentle stirring. The mixture was poured into molds and incubated at 40 °C for 2 h to induce chemical cross-linking, then cooled to 25 °C for physical cross-linking, and stored overnight at 25 °C in sealed molds. The three oxidized polysaccharide hydrogels (GE-Os) with different cross-linking degrees (GE-O1, GE-O2, GE-O3) were thus obtained.

#### 4.4.1. Characterization of Oxidized ARP and Polysaccharide Hydrogels

Infrared spectra of the oxidized samples were recorded using an IRAffinity-1 Fourier transform infrared spectrometer (Shimadzu, Kyoto, Japan) in the 4000–400 cm^−1^ range with the KBr-disk method. Surface morphology was examined using a field-emission scanning electron microscope (FEI Quanta 250, Thermo Fisher Scientific, Waltham, MA, USA; 15 kV accelerating voltage) and a Bruker ICON atomic force microscope (Dimension ICON, Bruker, Billerica, MA, USA). Spatial structure was analyzed by the Congo red method, which detects triple-helix conformations based on the red shift in the maximum absorption wavelength (λ_max_) in NaOH solutions of varying concentrations [43].

#### 4.4.2. Determination of the Degree of Cross-Linking

The cross-linking degree of GE-Os was evaluated using the ninhydrin colorimetric method [44]. A 2% (*w*/*v*) ninhydrin solution was freshly prepared and stored under light-protected conditions. Glycine solutions at graded concentrations (0–0.5 mg/mL) were used as calibration standards. For sample analysis, 2 mg of GE-O1, GE-O2, or GE-O3 were individually dissolved in 1 mL of deionized water, combined with 1 mL of ninhydrin reagent (2%, *w*/*v*), and mixed thoroughly. The mixtures were heated in a 100 °C water bath for 20 min in the dark and then cooled to room temperature. Absorbance was measured at 570 nm using a UV-vis spectrophotometer (P9, Shanghai Mapada Instruments Co., Ltd., Shanghai, China). The free amino group content was quantified based on the glycine calibration curve and the cross-linking degree was calculated using Equation (2):

Cross-linking degree (%) = (1 − N_t_/N_0_) × 100 (2)

(where N_0_ and N_t_ represent the free amino group contents before and after cross-linking, respectively).

#### 4.4.3. Injectability and Rheology Analysis

Injectability of GE-Os was assessed by loading each hydrogel into a 1 mL disposable syringe and extruding it through a 22-gauge needle with an internal diameter of 0.45 mm. The rheological behavior of GE-Os was measured using a rotational rheometer (MCR102e, Anton Paar, Graz, Austria). The storage modulus (G′) and loss modulus (G″) were recorded across strain levels from 0.1% to 100% at a fixed frequency (1.0 Hz) at room temperature.

#### 4.4.4. Mechanical Properties

Mechanical properties of GE-Os were assessed using a low-load tensile tester (UTM-4103, Jinan Kesheng Testing Equipment Co., Ltd., Jinan, China). Compressive properties of strength were quantified on freshly prepared hydrogels (2 mm × 1.5 mm × 4 mm) at room temperature with a displacement rate of 2 mm/min.

#### 4.4.5. Swelling and Degradation Properties

The swelling behavior of the three oxidized polysaccharide hydrogels (GE-Os) was analyzed by recording weight changes before and after incubation in PBS buffer at 37 °C. The hydrogels were measured at intervals of 0, 1, 2, 4, 6, 8, 12, and 24 h. The swelling ratio was calculated according to Equation (3).

Swelling ratio = [(m_t_ − m_0_)/m_0_] × 100% (3)

In vitro degradation of the GE-Os was performed by weight loss measurement in PBS solution. Hydrogels at swelling equilibrium were incubated at 37 °C, removed at specific time intervals (0, 1, 2, 4, 8, 16, 24, 36, 48 h), and weighed (W_t_) after gently blotting excess water with filter paper. The degradation rates of the hydrogels at different time points were calculated according to Equation (4), and the degradation curves were plotted with time and degradation rate to analyze the degradation kinetics of the hydrogels.

Degradation rate = (W_t_/W_0_) × 100% (4)

(where m_0_ and W_0_ are the initial mass of the hydrogel and m_t_ and W_t_ are the mass after immersion for different time intervals).

#### 4.4.6. Molecular Dynamics Simulation of Hydrogels

Molecular dynamics (MD) simulation is a widely employed computational approach for elucidating microscopic mechanisms underlying experimental observations. Based on Newtonian mechanics, MD simulates molecular motion to quantify thermodynamic properties and other structural features of a system [45]. To further elucidate the formation mechanism of oxidized polysaccharide hydrogels, MD simulations were performed using the GROMACS package (version 2020.6) [46] with the Generalized Amber Force Field (GAFF2) and TIP3P water model. Twenty random copolymer chains of dialdehyde arabinogalactan (1:3 ratio) were randomly placed in an 8 × 8 × 8 nm^3^ simulation box, representing the hydrogel framework. Subsequently, 280 short amine chains were randomly inserted, followed by solvation with over 10,000 water molecules. After several thousand steps of energy minimization, the system reached equilibrium under an isothermal–isobaric (NPT) ensemble, followed by a 50 ns production run. Temperature was controlled at 298 K using the Nose–Hoover thermostat, and pressure was maintained at 1 atm using the Parrinello–Rahman barostat. For non-bonded interactions, a 1.2 nm truncation scheme was applied, and long-range electrostatic interactions were computed using the particle–mesh Ewald method with a Fourier spacing of 0.1 nm [47]. The LINCS algorithm [48] was used to constrain all covalent bonds containing hydrogen atoms. The presence of hydrogen bonds was calculated using the commonly used criterion that the maximum hydrogen donor–acceptor angle is 30 degrees and the distance between the acceptor and donor atoms is less than 0.35 nm [49].

### 4.5. Preparation of CpG ODN–Loaded Hydrogel Complexes

During hydrogel preparation, 1 mM of CpG ODN 1862 solution was added dropwise to 10 mL of oxidized ARP hydrogel solution to allow for electrostatic adsorption. The mixture was poured into molds and incubated at 40 °C for 2 h to induce chemical cross-linking, followed by cooling to 25 °C to promote physical cross-linking. The resulting hydrogels were stored overnight at 25 °C in sealed molds, yielding three drug-loaded complexes: GE-O1-CpG, GE-O2-CpG, and GE-O3-CpG. Loading efficacy was calculated by quantifying residual CpG ODNs in the supernatant after 30 min of equilibrium, as described in Supplementary Materials File S2.

### 4.6. Zeta Potential of Hydrogel Complexes

The drug-loaded hydrogel complexes were dissolved in deionized water and vortexed or sonicated (5–10 min) to ensure even dispersion of hydrogel particles/molecules and prevent agglomeration. The prepared samples were then injected into clean cuvettes, carefully avoiding air bubbles, and placed in a Zeta Potentiostat (Zetasizer Nano S90, Malvern Panalytical, Malvern, UK) for five measurement cycles.

### 4.7. In Vitro Immunomodulatory Activity of Oxidized ARP and Hydrogels

#### 4.7.1. RAW 264.7 Macrophage Proliferation

A Cell Counting Kit-8 assay (CCK-8) was used to evaluate cell proliferation. Logarithmically growing cells were diluted to 1 × 10^5^ cells/mL, and 100 μL of this suspension was seeded into each well of a 96-well plate, followed by incubation at 37 °C with 5% CO_2_ for 24 h. For the experimental groups, the three oxidized polysaccharide (O1-ARP, O2-ARP, and O3-ARP) solutions were added at final concentrations of 5, 40, 60, 80, and 120 μg/mL, along with GE-O extracts from the three oxidized ARP hydrogels. Each condition was tested in triplicate. Plates were incubated for 36 h. The positive control group was treated with LPS at 2.5 μg/mL. After 36 h, culture supernatants were removed, and 100 μL of medium containing 10% CCK-8 solution was added to each well. After 1 h of incubation, absorbance at 450 nm was recorded using a microplate reader. Cell proliferation was calculated using Equation (5).

(5) $$\mathrm{Cell}\;\mathrm{viability}\left(\%\right)=\frac{A_{1}-A_{0}}{A_{2}\;-A_{0}}\;\times \;100\%$$

(where A_1_ is the experimental group (containing cell culture medium, CCK-8, and polysaccharide solution), A_2_ is the control group (medium + CCK-8 without polysaccharide), and A_0_ is the blank group (medium without cells and polysaccharide, plus CCK-8)).

#### 4.7.2. TNF-α, IL-1β, and IL-6 Cytokine and NO Production

RAW 264.7 cells were seeded into 96-well plates at 1 × 10^6^ cells/well and treated with oxidized polysaccharides (12.5–200 μg/mL) or GE-O extracts for 48 h. Cells cultured in medium alone served as the blank control, while those treated with 2.5 μg/mL LPS served as the positive control. After incubation, culture supernatants were collected, and the levels of IL-6, TNF-α, IL-1β, and NO were quantified using precoated mouse ELISA kits (Dakewei Bioengineering Co., Ltd., Shenzhen, China) according to the manufacturer’s protocols.

#### 4.7.3. Phagocytic Activity of RAW 264.7 Cells

Phagocytic activity was determined using the neutral red uptake method. Log-phase RAW 264.7 cells were suspended at 4 × 10^5^ cells/mL, and 100 μL of suspension was introduced into each well of a 96-well plate, followed by incubation at 37 °C with 5% CO_2_ for 24 h. For controls, untreated cells served as the negative group, while LPS (2.5 μg/mL) was added to the positive group. Experimental groups received oxidized polysaccharides (O1-ARP, O2-ARP, O3-ARP) at 5, 40, 60, 80, and 120 μg/mL, or GE-O extracts. Each condition was tested in triplicate and incubated under identical conditions for 24 h. After incubation, the supernatant was discarded and cells were washed twice with PBS. Then, 100 μL of medium containing 10% neutral red solution was added and incubated for 1 h. Cells were again washed with PBS and lysed with 100 μL of cell lysate, followed by 2 h of incubation. Optical density (OD) was measured at 540 nm using a microplate reader, and the macrophage phagocytic index was calculated using Equation (6):

(6) $$\mathrm{PI}\left(\%\right)\;=\;\frac{A_{1}}{A_{2}}\;\times \;100\%$$

(where A_1_ is the experimental group and A_2_ is the control group (polysaccharide-free solution)).

### 4.8. In Vitro Immunomodulatory Activity of Hydrogel Complexes

GE-O1, which exhibited superior immunomodulatory activity, was selected as the potential drug carrier. IL-6, TNF-α, and NO levels after stimulation with GE-O1 and the drug-loaded hydrogel complex (GE-O1-CpG) were evaluated. The experimental procedure followed the same method as described in Section 4.7.2. TNF-α, IL-1β, and IL-6 Cytokine and NO Production.

### 4.9. Cellular Uptake Assay of Hydrogel Complexes

To further examine the uptake of GE-O1-CpG by RAW 264.7 macrophages, 5 × 10^4^ RAW 264.7 cells/well were seeded into 24-well plates, with 500 μL of cell suspension added to each well, and incubated for 24 h until full adherence. The medium was then removed, and the cells were gently washed twice with PBS. The experimental groups included varying concentrations of GE-O1 with FAM-ODN 1862; the control group received only fresh medium, and the blank hydrogel control group contained GE-O1 without FAM-ODN 1862. The 24-well plates were incubated for 1 h, followed by staining with DAPI (4′,6-diamidino-2-phenylindole). Fluorescence staining of the cells was observed using a fluorescence microscope (Olympus IX73, Olympus Corporation, Tokyo, Japan).

### 4.10. Drug Release Performance of Hydrogel Carriers

Briefly, 1 mL of GE-O1-CpG was placed into physiological PBS, acidic PBS (pH = 5.4), and α-glucosidase solution (pH = 5.4), respectively. Each group was incubated in a 37 °C constant-temperature shaker at 100 r/min for 30 min to allow for pre-equilibration and reach initial balance. At 0 h (initial sample), 0.5 h, 1 h, 2 h, 4 h, 8 h, 12 h, 24 h, and 48 h, 30 μL of solution was withdrawn from each tube, transferred to a new EP tube, and centrifuged, and 30 μL of the supernatant was collected. The concentration of free CpG ODN 1862 was measured using a NanoDrop 2000 spectrophotometer (Thermo Fisher Scientific Inc., USA). To calculate the cumulative release amount of CpG ODN 1862 at each time point, the following equation was used:

The cumulative release amount (μg) = measured concentration (μg/mL) × sampling volume (mL).

### 4.11. Hemocompatibility

Blood samples were collected from naïve mice, put into anticoagulant tubes and centrifuged at 1200 rpm for 5 min. The supernatant was removed and erythrocytes were collected. A 2% hematocrit erythrocyte suspension was prepared in PBS. OVA and each adjuvant (F/OVA, CpG/OVA, CpG/OVA) were mixed with the erythrocyte suspension (4:1, *v*/*v*) and incubated statically at 37 °C for 1 h. After centrifugation (1200 rpm, 3 min), the supernatant was collected and 150 µL aliquots were transferred into a 96-well plate. Absorbance was measured at 540 nm. The hemolysis rate was calculated using Equation (7):

(7) $$\mathrm{Hemolysis}\left(\%\right)\;=\;\frac{{OD}_{1}\;-\;{OD}_{0}}{{OD}_{2}\;-\;{OD}_{0}}\;\times \;100\%$$

(where OD_1_ is the experimental group (sample + erythrocytes), OD_2_ is the positive control (deionized water + erythrocytes), and OD_0_ is the blank control (saline + erythrocytes)).

### 4.12. Adjuvant Activity Assays In Vivo

#### 4.12.1. Animal Immunization

All animal experimental procedures followed the Guidance on the Operation of the Animals (Scientific Procedures) Act 1986 and the NIH (National Research Council) Guide for the Care and Use of Laboratory Animals, and were approved by the Animal Use and Care Committee of Inner Mongolia Medical University, China (Permit No: YKD202101170). Five-week-old ICR mice (*n* = 24/group) with males and females in equal proportions were randomly assigned to five groups: PBS control, OVA (50 μg), F/OVA (50 μg OVA + 100 μg Freund), GE-O1-CpG/OVA (50 μg OVA + 65 μg GE-O1-CpG), and CpG/OVA (50 μg OVA + 65 μg CpG)/mouse. Mice received 0.2 mL subcutaneous injections in the dorsal region on days 0 (primary), 7 (secondary), and 14 (tertiary immunization).

#### 4.12.2. Determination of Immune Organ Index

On days 7, 14, and 21 post-immunization, three mice per group were randomly euthanized. Blood was collected via retro-orbital puncture prior to euthanasia. Body weight was recorded, followed by dissection and isolation of spleens and thymuses. Organ weights were measured, and indices were calculated as follows:

Spleen index (mg/g) = Spleen mass (mg)/Body weight (g)

Thymus index (mg/g) = Thymus mass (mg)/Body weight (g)

#### 4.12.3. Splenic Lymphocyte Proliferation

Splenocytes were isolated from three mice per group on days 7 and 28 post-immunization. Mice were euthanized by cervical dislocation and surface-sterilized in 75% ethanol. Spleens were aseptically excised and mechanically dissociated through a cell strainer into PBS. Suspensions were centrifuged at 1200 rpm for 3 min, and the resultant pellets were treated with 3 mL of erythrocyte lysis buffer for 5 min. Lysis was neutralized with twice the volume of RPMI-1640 medium. Cells were pelleted, washed twice with PBS, and resuspended in complete RPMI-1640 medium. Cell concentrations were adjusted to 1 × 10^6^ cells/mL in OVA-containing medium (50 μg/mL). Splenocytes were seeded at 1 × 10^6^ cells/well in 96-well plates and stimulated with PHA (2.5 μg/mL) or LPS (1 μg/mL). Wells containing serum-free RPMI-1640 served as blanks. All conditions were tested in quadruplicate. After 48 h of incubation at 37 °C, 5% CO_2_, 20 μL of CCK-8 reagent was added per well. Absorbance at 450 nm was recorded 4 h later. Lymphocyte proliferation was expressed as stimulation index (SI) or OD values according to Equation (8).

(8) $$\mathrm{Cell}\;\mathrm{viability}(\%)=\frac{{OD}_{1}\;-\;{OD}_{0}}{{OD}_{2}\;-\;{OD}_{0}}\;\times \;100\%$$

(where OD_1_ is the experimental group (cell culture medium + CCK-8 + polysaccharide solution), OD_2_ is the control group (cell culture medium + CCK-8 without polysaccharide), and OD_0_ is the blank group (medium without cells or polysaccharide, plus CCK-8)).

#### 4.12.4. OVA-Specific IgG Antibody Analysis—IgG, IgG1, IgG2a

To assess antibody secretion, blood samples were collected on days 7, 14, and 21 after the primary immunization. Serum was separated by centrifugation at 1200× rpm for 5 min. OVA-specific IgG, IgG1, and IgG2a concentrations were quantified using established methods (*n* = 4).

#### 4.12.5. ELISA Analysis of Cytokine Levels in Serum Lymphocytes

On day 14, three mice per group were randomly selected. After terminal retro-orbital blood collection, samples were transferred into clot activator tubes. Serum was obtained by centrifugation at 3000× rpm for 30 min, and the pale-yellow upper layer was collected. Concentrations of IL-4, IFN-γ, IL-17, and TNF-α were determined by ELISA.

#### 4.12.6. Biosafety Analysis

On day 28 post-primary-immunization, serum and major organs (heart, liver, spleen, lungs, and kidneys) were extracted from each group. Organs were fixed in 4% paraformaldehyde and stained with hematoxylin and eosin (H&E). Histopathological morphology was investigated under a light microscope. Serum biochemical parameters were measured with an automated analyzer, including aspartate aminotransferase (AST), alanine aminotransferase (ALT), alkaline phosphatase (ALP), lactate dehydrogenase (LDH), and blood urea nitrogen (BUN).

### 4.13. Statistical Analysis

Experimental data were expressed as mean ± standard deviation (SD) and analyzed using GraphPad Prism 10 software. Differences between groups and versus the blank control were assessed by ANOVA. Statistical significance was set at *p* < 0.05, *p* < 0.01, and *p* < 0.001 compared with the control group.

## Figures and Tables

**Table 1:** Chemical compositions of ARP and oxidized derivatives.

Items	ARP	O1-ARP	O2-ARP	O3-ARP
Total carbohydrate (%)	91.00	60.34	67.28	70.13
Molecular weight (Da)	1.21 × 10^4^	0.6468 × 10^4^	0.7627 × 10^4^	0.9928 × 10^4^
Aldehyde concentrations (mol/g)	----	0.3260	0.2188	0.1900
Fucose	8.930	3.400	3.160	8.275
Rhamnose	20.66	5.520	5.700	15.51
Arabinose	59.26	60.01	59.23	56.73
Galactose	124.6	21.75	22.64	84.50
Glucose	74.51	9.700	10.03	28.43
Mannose	40.14	3.740	4.100	19.58
Xylose	7.400	2.540	2.550	3.675
Galacturonic acid	66.26	6.260	7.060	72.55
Glucuronic acid	13.57	0.00	0.00	1.639

## Data Availability

The original contributions presented in this study are included in the article/Supplementary Materials. Further inquiries can be directed to the corresponding authors.

## Supplementary Materials

The following supporting information can be downloaded at: https://www.mdpi.com/article/10.3390/gels12080703/s1, Figure S1: Ion chromatogram. (A) Ion chromatogram of ARP mixed with monosaccharides; (B) Ionic chromatogram of O1-ARP mixed with monosaccharides; (C) Ionic chromatogram of O2-ARP mixed with monosaccharides; (D) Ionic chromatogram of O3-ARP mixed with monosaccharides; Figure S2: Cross-linking degree of CE-Os. Glycine standard curve (left); cross-linking degree of CE-Os (right); Figure S3: Molecular dynamics (MD) simulation mechanism of GE-Os. (A) Electrostatic energy (Coulomb force) and van der Waals energy in intermolecular interactions. (B) Electrostatic energy (Coulomb force) and van der Waals energy in water molecules. (C) SASA profile with respect to the duration of the simulation; Figure S4: Zeta potential of CE-Os and the drug-loaded hydrogel complexes; Figure S5: (A) The change of body weight in male. (B) The change of body weight in female; Figure S6: Organ Indexes of the major organs on day 15 post-booster, (A) The heart (B) The liver (C) The kidney (D)The lung (E) The spleen; Figure S7: Levels of AKP, ALT, AST, BUN, and LDH in serum; Figure S8: H&E staining images of the major organs measured on day 28; Supplementary Materials File S1: Injectability video of hydrogel; Supplementary Materials File S2: The loading efficacy of the three oxidized polysaccharide hydrogels; 1. The content of total sugar and protein 2. Molecular weights determination 3. Monosaccharide composition.
