# Supplementary material for: Injectable Arnebia Euchroma Polysaccharide-Based Hydrogel as CpG Oligonucleotide Delivery System with Dual Immunomodulatory Activities
Source: Gels. 2026 Aug 5;12(8):703. doi: 10.3390/gels12080703 (PMC13512123; doi:10.3390/gels12080703)
Supplement: Supplementary file 1 [file gels-12-00703-s001.zip › Figure S1.pdf]

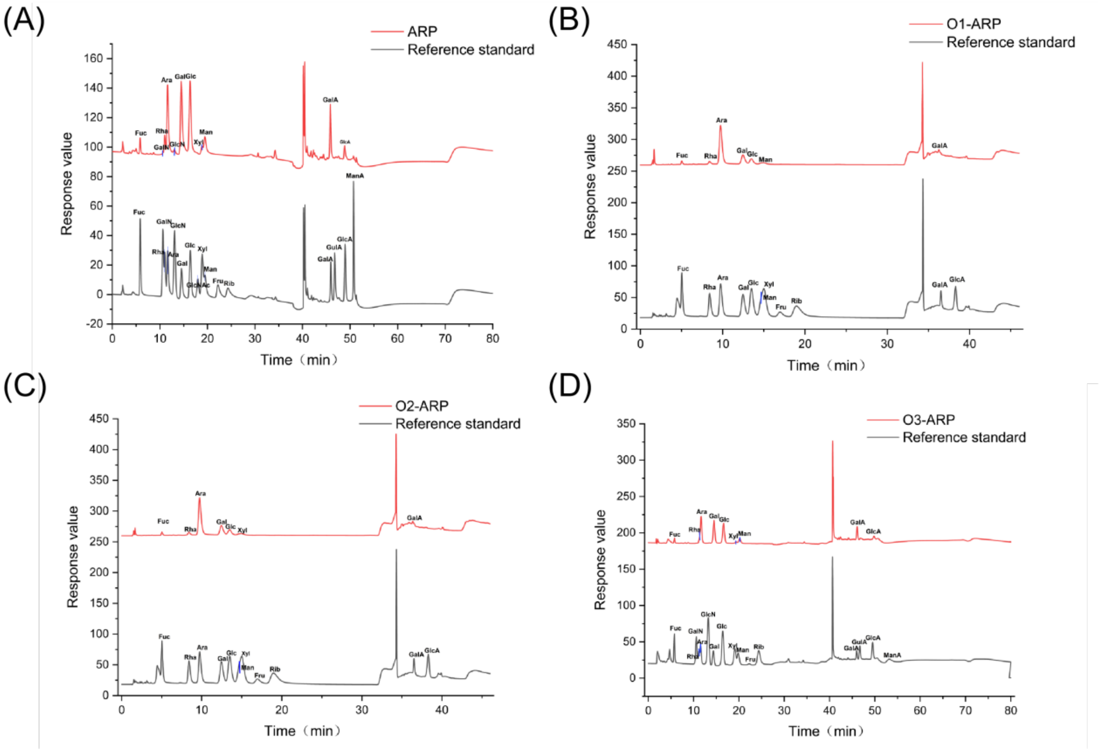

Figure S1. Ion chromatogram. (A) Ion chromatogram of ARP mixed with monosaccharides; (B) Ionic chromatogram of O1-ARP mixed with monosaccharides; (C) Ionic chromatogram of O2-ARP mixed with monosaccharides; (D) Ionic chromatogram of O3-ARP mixed with monosaccharides
