# Supplementary material for: Injectable Arnebia Euchroma Polysaccharide-Based Hydrogel as CpG Oligonucleotide Delivery System with Dual Immunomodulatory Activities
Source: Gels. 2026 Aug 5;12(8):703. doi: 10.3390/gels12080703 (PMC13512123; doi:10.3390/gels12080703)
Supplement: Supplementary file 1 [file gels-12-00703-s001.zip › Figure S3..pdf]

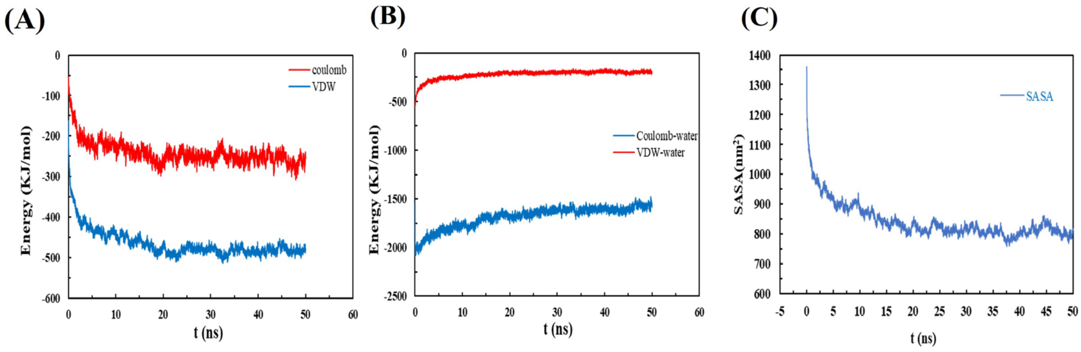

Figure S3. Molecular dynamics (MD) simulation mechanism of GE-Os. (A) Electrostatic energy (Coulomb force) and van der Waals energy in intermolecular interactions. (B) Electrostatic energy (Coulomb force) and van der Waals energy in water molecules. (C) SASA profile with respect to the duration of the simulation.
