# Supplementary material for: Injectable Arnebia Euchroma Polysaccharide-Based Hydrogel as CpG Oligonucleotide Delivery System with Dual Immunomodulatory Activities
Source: Gels. 2026 Aug 5;12(8):703. doi: 10.3390/gels12080703 (PMC13512123; doi:10.3390/gels12080703)
Supplement: Supplementary file 1 [file gels-12-00703-s001.zip › Figure S6.pdf]

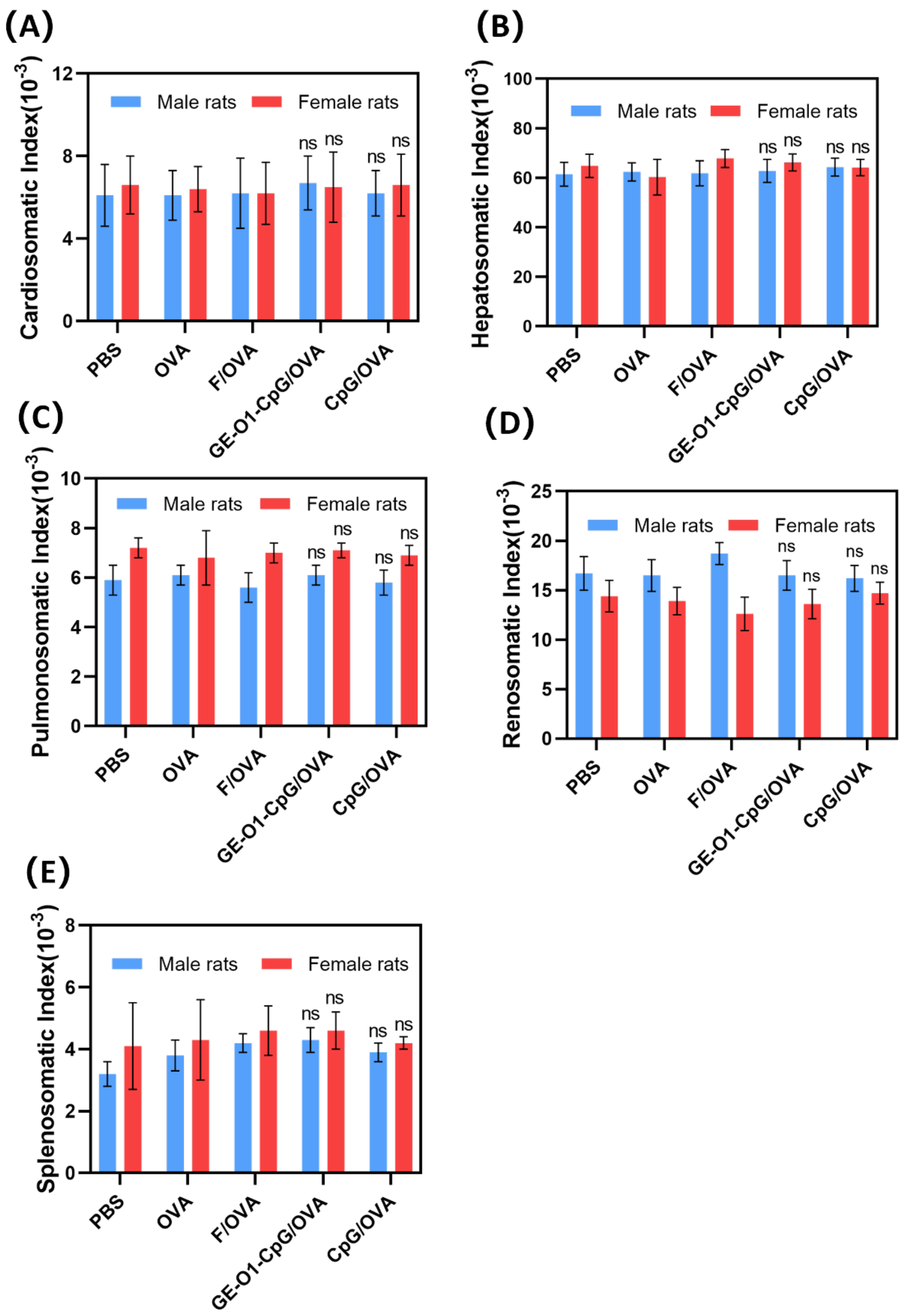

Figure S6. Organ Indexes of the major organs on day 15 post-booster,

(A) The heart (B) The liver (C) The kidney (D)The lung (E) The spleen.
