# Supplementary material for: Injectable Arnebia Euchroma Polysaccharide-Based Hydrogel as CpG Oligonucleotide Delivery System with Dual Immunomodulatory Activities
Source: Gels. 2026 Aug 5;12(8):703. doi: 10.3390/gels12080703 (PMC13512123; doi:10.3390/gels12080703)
Supplement: Supplementary file 1 [file gels-12-00703-s001.zip › Figure S8..pdf]

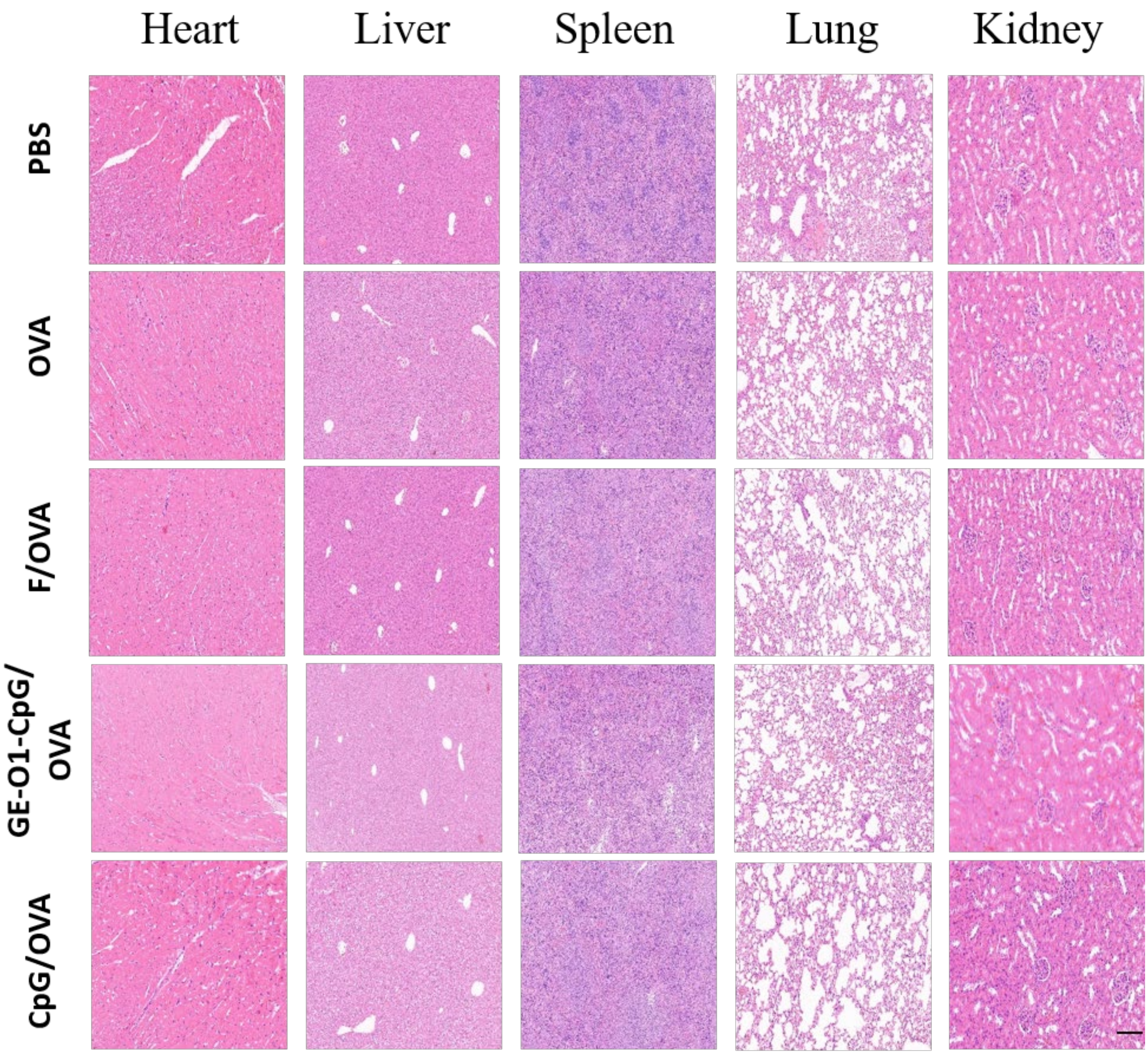

Figure S8. H&E staining images of the major organs measured on day 28.
