# Supplementary material for: Injectable Arnebia Euchroma Polysaccharide-Based Hydrogel as CpG Oligonucleotide Delivery System with Dual Immunomodulatory Activities
Source: Gels. 2026 Aug 5;12(8):703. doi: 10.3390/gels12080703 (PMC13512123; doi:10.3390/gels12080703)
Supplement: Supplementary file 1 [file gels-12-00703-s001.zip › Supplementary Materials File S2.pdf]

The loading efficacy of the three oxidized polysaccharide hydrogels

The loading capacity of CpG oligonucleotide drugs in the hydrogel system was calculated based on the initial concentration of the nucleic acid drug and the concentration of encapsulated drug. The encapsulated drug concentration was determined by subtracting the concentration of free nucleic acids in the supernatant from the initial concentration. The loading capacity was then obtained by dividing the initial drug concentration by the encapsulated drug concentration.

For example:

The initial concentration of the nucleic acid drug= 32.40 μg/mL

The concentration of free nucleic acids for GE-O1=11.74μg/mL

The loading capacity of GE-O1= 32.4-117.4/32.4 *100%= 63.4%.
