# Supplementary material for: Injectable Arnebia Euchroma Polysaccharide-Based Hydrogel as CpG Oligonucleotide Delivery System with Dual Immunomodulatory Activities
Source: Gels. 2026 Aug 5;12(8):703. doi: 10.3390/gels12080703 (PMC13512123; doi:10.3390/gels12080703)
Supplement: Supplementary file 1 [file gels-12-00703-s001.zip › Supplementary Materials File S3.pdf]

Supplementary methods

1. The content of total sugar and protein

The sugar levels were measured using the phenol sulfuric acid technique by UV-Vis spectrometer at 490 nm.

2. Molecular weights determination

Using our previously published approach, high performance gel permeation chromatography (HPGPC) was used to measure the average molecular weight of the oxidized polysaccharides, in combination with a HPLC system (LC-10A) equipped with a SUGAR BRT 105-103-101 (8mm x 250 mm). The calibration curve was drawn by standards of dextran with varying molecular weights (1,153, 6,941, 9,835, 20,600, 47,880, 112,200, 240.100, 396,400, 883,100 and 1689,322 Da). The 5 mg of the oxidized derivatives were dissolved in mobile and filtered for further analysis.

3. Monosaccharide composition

The sugar composition of the ARP and three oxidized polysaccharides was found using ion chromatography according to previous reported method with 11 kinds of monosaccharides as the standard for qualitative and quantitative analysis.

ARP and O-ARPs (4 mg) underwent hydrolysis with 2M trifluoroacetic acid (TFA) in a sealed tube at 120°C for three hours. 200 μL of the acid hydrolysis solution was transferred to a 1.5 ml EP tube, and blew dry by Nitrogen gas. The Residue vortexed with 1 ml of water, and centrifuged at 12000 rpm for 5 min. The supernatant was taken into IC for analysis.
